# Are cell length and volume interchangeable in cell cycle analysis?

**DOI:** 10.1016/j.bpj.2025.03.019

**Published:** 2025-03-26

**Authors:** Prathitha Kar, Ariel Amir

**Affiliations:** 1 Department of Chemistry and Chemical Biology, Harvard University, Cambridge, Massachusetts; 2 School of Engineering and Applied Sciences, Harvard University, Cambridge, Massachusetts; 3 Department of Physics of Complex Systems, Weizmann Institute of Science, Rehovot, Israel

## Abstract

Cell length has been used as a proxy for cell size in cell cycle modeling studies. A previous study, however, brought into question the validity of this assumption, noting that correlations between cell lengths can be different from those involving cell volume if cell width fluctuations are taken into account. If cell volume is regulated, data analysis involving cell lengths will lead to an incorrect inference of the cell size control mechanism. We used conditional correlation of length variables conditioned upon radius variables to elucidate the underlying volume control mechanism. Using the conditional correlation on previous mother machine datasets measuring lengths at birth and division and the cell radius for multiple cells, we find that the cell volume control strategy is consistent with an adder model. Further, using the conditional correlation, we conclude that measurement noise constitutes a significant portion of the radius variability in the experimental datasets. To conclude, cell length and cell volume can often be used interchangeably owing to small cell width fluctuations.

## Significance

Cell cycle studies regularly use correlations between cell lengths at various cell cycle checkpoints to elucidate the underlying mechanisms. It is unclear whether length is a good proxy for cell volume. Indeed, studies in fission yeast showed that cell width fluctuations can influence the measured correlations and make length and volume noninterchangeable. Using modeling and data analysis of *Escherichia coli* experimental data, we find that cell width fluctuations have a negligible impact on the correlation structure of cell cycle variables. This implies that for bacteria such as *E. coli*, length and volume can often be used interchangeably. Our analysis suggests that cell width is tightly regulated in *E. coli* to an accuracy of ≈4% or 10 nm.

## Introduction

**Figure 1:**
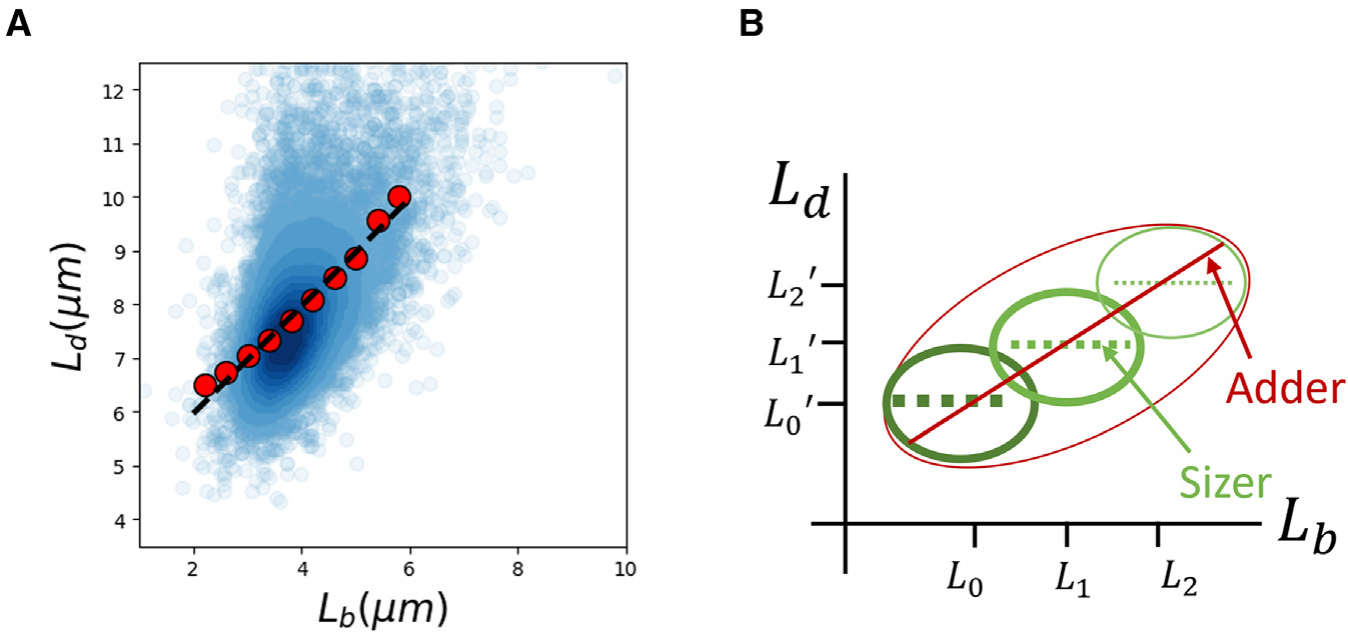
(*A*) An example where the adder model of cell division, in which cells divide upon adding a constant size from birth, is consistent with the experimental data of *E. coli*. The blue cloud is the raw data obtained from (6) growing in a growth medium with the average doubling time $\left(\left\langle T_{d}\right\rangle \right)=17$ min (no. of cells *n* = 18,248). The red dots are the experimental binned data trend calculated by dividing the *x* axis values into bins of equal width and calculating the mean of the *y* axis values in that bin. The binned data trend matches the prediction for the binned data trend of the adder model (*black dashed line* with slope = 1). (*B*) Illustration of Simpson’s paradox affecting length correlations. The underlying model could be a volume sizer with width fluctuations. Each green cloud corresponds to cells of different radii and is a volume sizer. Each subpopulation with a different radius is also a length sizer but with different average birth and division lengths. When the population of cells of different radii is combined, the correlation resembles that of a length adder.

Recent developments in microscopy and microfluidics have enabled researchers to study cell cycle regulation at a single-cell level (1). Data analysis methods and quantitative models complement these experiments, allowing us to make progress in our understanding of cell size homeostasis. Coarse-grained phenomenological models agnostic of the molecular details have provided clues about cell size regulation mechanisms (2,3,4). Often, the Pearson correlation coefficient and best linear fit predictions from models are compared against experimental data to uncover the underlying biological mechanism. We show an example (Fig. 1 *A*) where the adder model of cell division—cells divide upon adding a constant size from birth (*black dashed line*)—is consistent with the binned data (*red points*) from experiments on *Escherichia coli* (5,6,7,8,9). Throughout this paper, binned data are calculated by dividing the *x* axis values into equal-width bins and finding the mean of the *y* axis values. In this analysis, cell length is assumed to be a proxy for cell volume, and they are often used interchangeably.

(10) argued that they are not interchangeable cell characteristics. Their logic followed a scenario pointed out in (2). (10) discussed that subpopulations with different mean lengths at birth and division can arise despite carefully maintained growth conditions in microfluidics experiments due to natural cell width variability. For a population of cells with multiple subpopulations differing in their average length at birth and division, a division strategy called the sizer model, where cells divide upon reaching a critical size (slope = 0 in the $L_{d}$ versus $L_{b}$ plot in each subpopulation) can be misconstrued as an adder (slope = 1 in the $L_{d}$ versus $L_{b}$ plot) when the whole population is considered (Fig. 1 *B*). This is the so-called “Simpson’s paradox” and, in this case, leads to the actual sizer model being interpreted as an adder when various subpopulations are analyzed together. They found that cell width fluctuations could explain the positive length correlations observed in experimental data on fission yeast despite each subpopulation being a sizer. Using the same argument for *E. coli*, they found that a volume sizer, along with cell width fluctuations, could lead to a slope = 1 for the best linear fit of the $L_{d}$ versus $L_{b}$ plot. The underlying biological mechanisms leading to a sizer differ from those resulting in an adder. This would mean that cell cycle analyses involving cell length data require width fluctuations to be accounted for. An important thing to note is that the measured cell radius variability is small (≈6%) (6). However, calculations in (10) and this paper show that the slope of $L_{d}$ versus $L_{b}$ is dependent on the ratio of radius variability and the noise in setting the division volume. Since the division volume noise is also small, there could be a substantial difference between the slope calculated using the entire population of cells and each subpopulation, even for a small radius variability.

In this paper, we discuss various cell division models that take cell width fluctuations into account. First, we show that the model proposed in (10) is inconsistent with experimental data from *E. coli* regarding the correlation between birth lengths in successive generations. Based on a general model of cell division and cell width fluctuations, we devise a method involving conditional correlation of length variables conditioned upon radius variables to elucidate the underlying division volume control mechanism. Upon accounting for the width fluctuations, we find *E. coli* experimental data to be consistent with an adder model of cell division. Using conditional correlations, we also estimate that the actual radius variability is, at most, half of the measured variability, indicating that measurement noise is a major contributor to the radius measurements. This supports the assumptions from previous studies that cell lengths and volumes can be used interchangeably.

## Results and discussion

First, we will state the model proposed in (10) and obtain the correlation structure based on it. Using the model, we will restate the results in (10) and Fig. 1 *B* related to the correlation between the length at birth and division. Next, we will show that the minimal model fails to recover the correlations between birth lengths in successive generations.

**Figure 2:**
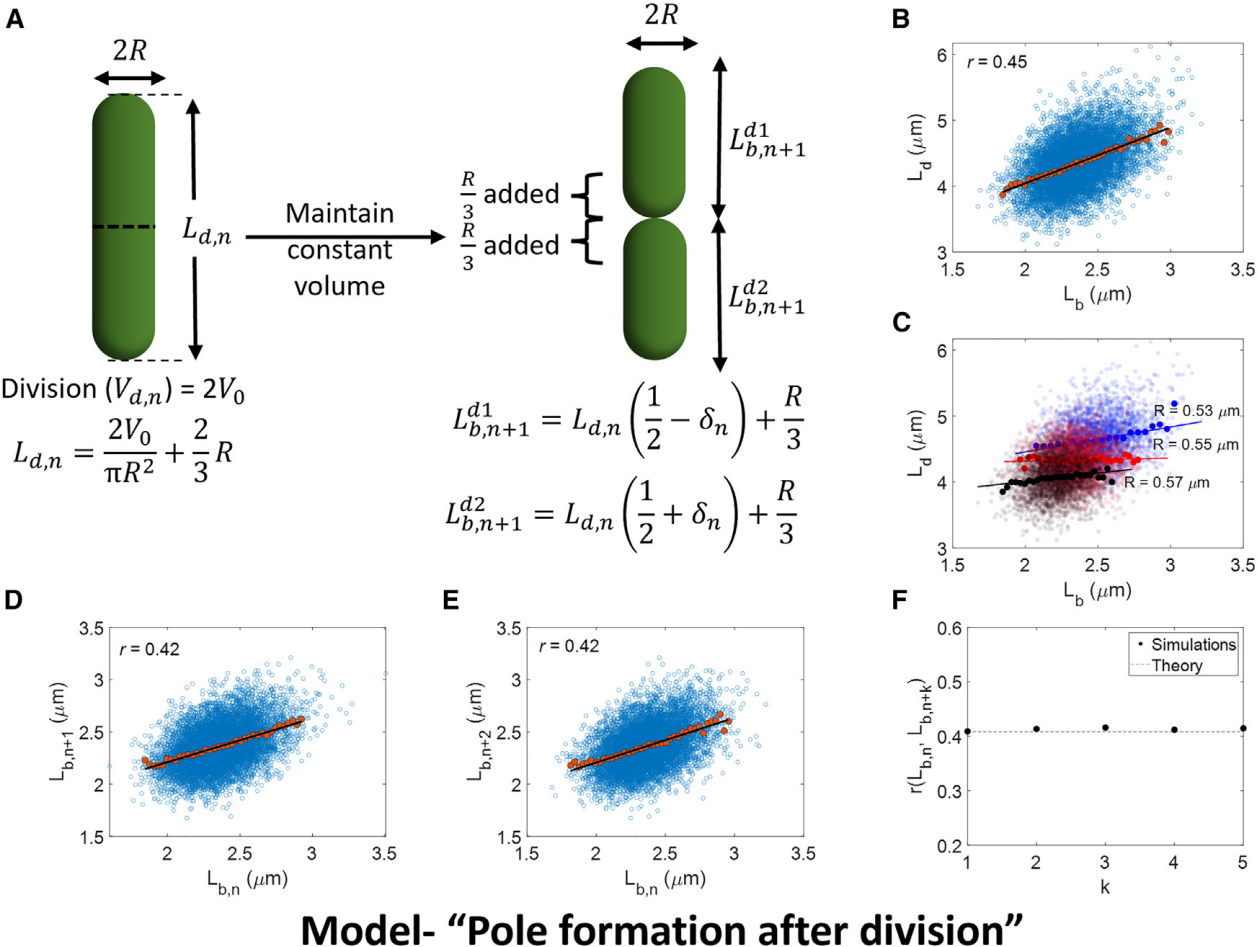
Model “pole formation after division.” (*A*) Schematic of the model proposed in (10). The cell divides when it reaches a critical volume 2 $V_{0}$ (volume sizer). The cells divide symmetrically by length on average, i.e., the mean division ratio = $\frac{1}{2}$ and the noise in division ratio for cells dividing in generation n = $\delta_{n}$. Upon dividing, the two daughter cells develop hemispherical poles at one end, rapidly keeping the total volume constant, i.e., the division volume of the mother cell is equal to the sum of the birth volumes of the two daughter cells. This amounts to an addition of $\frac{R}{3}$ term to the birth lengths. The cell radius is fixed for all cells in a particular lineage but varies for different lineages. (*B–E*) Simulations of the model in (*A*) are carried out for 10,000 cell lineages and over 25 generations. For the simulations, (*B*) we plot the $L_{d}$ versus $L_{b}$ plot. The correlation r (*top left*) points to a near-adder model. (*C*) We plot $L_{d}$ versus $L_{b}$ for small subsets of R. We arrange the simulated dataset in ascending order of radius and divide it into three groups. For each group (with a different average radius), we plot the $L_{d}$ versus $L_{b}$ plot. The plot shows the Simpson’s paradox mentioned in Fig. 1*B*. (*D*) Length at birth in generation n+1 versus generation n is plotted. (*E*) Length at birth in generation n+2 versus generation n is plotted. The correlation values are identical and consistent with Eq. 1. In all plots, the cloud is the raw data, the dots represent the binned data, and the line is the best linear fit. As is the case throughout the paper, binned data are calculated by dividing the *x* axis values into equal-width bins and taking the mean of the *y* axis values in those bins. (*F*) We calculate the correlation between the lengths at birth in generation n and k generations later for different k. Consistent with Eq. 1 and (*D*) and (*E*), we find the correlations to be constant independent of k.

In the model, *E. coli* single-cell geometry is approximated as a spherocylinder with identical cell radii (R) within a cell lineage (Fig. 2 *A*). However, in a typical cell cycle analysis such as the one in Fig. 1 *A*, the data combine cells from different lineages, which might have different cell widths (and equivalently radii). One scenario where such cell radius variation might arise is if the cell width varies at a timescale much larger than the doubling time of cells. In the subsequent section, we will discuss a model where the cell radius changes in consecutive generations of a cell lineage. We assume that the cell divides when it reaches a critical volume $2\;V_{0}$ (volume sizer). This is a special case of the general model where the volume at birth determines the cell division volume via a regulation strategy, $f\left(V_{b}\right)$ (11). A simple choice of $f\left(V_{b}\right)$ is $f\left(V_{b}\right)=2\left(1-\alpha \right)V_{b}+2\alpha V_{0}$, where α is the cell cycle regulation parameter. Mathematically, we can express the division volume to be $V_{d}$ = $2\left(1-\alpha \right)V_{b}+2\alpha V_{0}\left(1+\zeta_{s}\right)$, where $\zeta_{s}$ is the size additive division noise. α can assume any value between 0 and 2, with α = $\frac{1}{2}$ being the adder strategy and α = 1 the sizer strategy. The cells divide symmetrically on average, i.e., the mean division ratio = $\frac{1}{2}$ and the noise in division ratio for cells dividing in generation n = $\delta_{n}$. Upon dividing, the two daughter cells develop hemispherical poles at one end, keeping the total volume constant, i.e., the division volume of the mother cell is equal to the sum of the birth volumes of the two daughter cells. This amounts to an addition of $\frac{R}{3}$ term to the birth lengths (“pole formation after division” model in Fig. 2 *A*). (10) based the model on fission yeast growth, where a rapid increase in cell lengths is observed just after cell birth (12). A similar new pole formation pattern is observed in bacterial species such as *Bacillus subtilis* (13). In the next section, we will discuss a model of pole formation found in *E. coli* where cells start constricting and form the new hemispherical poles before division.

Upon simulating the model with the same model parameters as in (10), we recover their result of slope ≈1 for the best linear fit of the $L_{d}$ versus $L_{b}$ plot, which is consistent with a length adder (Fig. 2 *B*; supporting material section S1.1.1). As stated previously, this positive correlation comes from Simpson’s paradox (Fig. 1 *B*): consider cells with radii δR larger than the average. Such cells would have an average length at birth and division different from that of cells with radii δR smaller than average. Within each subpopulation, cells divide upon reaching a critical volume or equivalently critical length, as the cell width within the subpopulation is fixed (Fig. 2 *C*). Upon combining the different subpopulations, the shifts due to different length averages lead to an apparent length adder (Fig. 2 *B*).

Minimal cell division models that neglect width fluctuations are consistent with the experimental data regarding the correlation between cell birth lengths in successive generations. According to this model, the birth lengths n generations apart are correlated as ${\left(1-\alpha \right)}^{n}$ (4,11). (4) analyzed experimental data on *E. coli* from (14) and found the Pearson correlation coefficient between the birth lengths of mother and daughter cells to be approximately 0.5. The correlation between the birth lengths of mother and granddaughter cells was approximately 0.25. Together, these correlations point to α = 0.5, i.e., the adder model. We tested whether the model that includes width fluctuations also agrees with these correlations. Using the model, we found the correlation between length at birth at generation n $\left(L_{b,n}\right)$ and *k* generations later $\left(L_{b,n+k}\right)$ (supporting material section S1.1.2) to be

(1) $$r=\frac{4{\left(\frac{R_{0}}{3}-\frac{V_{0}}{\pi R_{0}^{2}}\right)}^{2}\sigma_{R}^{2}}{{\left(\frac{V_{0}}{\pi R_{0}^{2}}\right)}^{2}\sigma_{bd}^{2}+4{\left(\frac{R_{0}}{3}-\frac{V_{0}}{\pi R_{0}^{2}}\right)}^{2}\sigma_{R}^{2}+{\left(\frac{V_{0}}{\pi R_{0}^{2}}+\frac{R_{0}}{3}\right)}^{2}\sigma_{m}^{2}}\text{,}$$

where $R_{0}$ is the mean radius, $\sigma_{bd}$ is the standard deviation of division noise $\zeta_{s}$, $\sigma_{R}$ is the coefficient of variation (CV) in radius, and $\sigma_{m}$ is the standard deviation of division ratio $\delta_{n}$. Contrary to the decreasing correlations with increasing k that have been observed for experimental data (4), we found that correlations between successive birth lengths are constant and independent of k for this model (Eq. 1; Fig. 2, *D–F*). In this model, cell lengths have no memory of the prior generations (sizer) except for having the same radius. Since the radius stays constant within a lineage independent of k, the correlations do not vary despite considering cell lengths k generations apart. This is evident in Eq. 1, where the covariance between birth lengths k generations apart (the numerator in Eq. 1) is only dependent on the variability in cell width. The positive correlations in this case have the same origin as before, i.e., Simpson’s paradox (Fig. S1).

Thus, our analysis shows that a volume sizer with width fluctuations cannot account for the correlations between birth lengths observed in experimental data.

### Correlation structure in a cell division model where the new pole forms at mid-cell before division

In the previous model, hemispherical pole formation followed the cell division event. We wanted to verify that the timing of the new pole formation does not affect the correlation structure obtained from the model. The model in the previous section assumed a sudden increase in cell length in a small period around birth, which is not observed in *E. coli*. Therefore, we test a model of cell growth and division where cells constrict and form new poles before dividing (“pole formation before division” model).

The model borrows characteristics from the previous one, such as spherocylindrical cell geometry, constant radius along a lineage while variability exists across lineages, and a volume sizer. However, in this model, we assume that two spherocylindrical daughter cells are fully formed just before division. The two daughter cells are symmetrical on average, but there are fluctuations in the volume division ratio denoted by $\delta_{n}$. For an asymmetrical division in generation n, it is assumed that one of the cells receives an additional $\delta_{n}V_{d,n}$ in volume. However, the length partition for that cell is not $\delta_{n}L_{d,n}$ since cell length is a linear function of volume (Fig. S2 *A*). Partitioning by length does not impact the results qualitatively (Fig. S3, *A*–*C*).

We find that, for the same parameters as in the previous section, the Pearson correlation coefficient between $L_{b}$ and $L_{d}$ is close to 0.5, thus agreeing with a length adder (Fig. S2 *B*). It follows the same explanation as the previous section, where Simpson’s paradox leads to an apparent adder (Fig. S2 *C*). Importantly, the correlation between birth lengths, k generations apart, is a constant for k = 1 and 2 (Fig. S2, *D* and *E*; supporting material section S1.2). As was the case previously, the constancy in correlation k generations apart is because the lengths are correlated owing to the same radius in a lineage. Thus, the model fails in manner similar to that of the previous model.

### Correlation structure in a model where radius correlations between generations are not equal to one

Both previous models show that the volume sizer can lead to a length adder, but they do not agree with all the correlations observed in the experiments. The correlations between length births across generations do not decay because the radius in a lineage is fixed. However, if the cell width fluctuates on a timescale of cell doubling time, then the radius might be correlated but not equal to one between successive generations. In this section, we will discuss a model where we relax the assumption that the radius is fixed in a lineage (“changing radii in a lineage” model).

**Figure 3:**
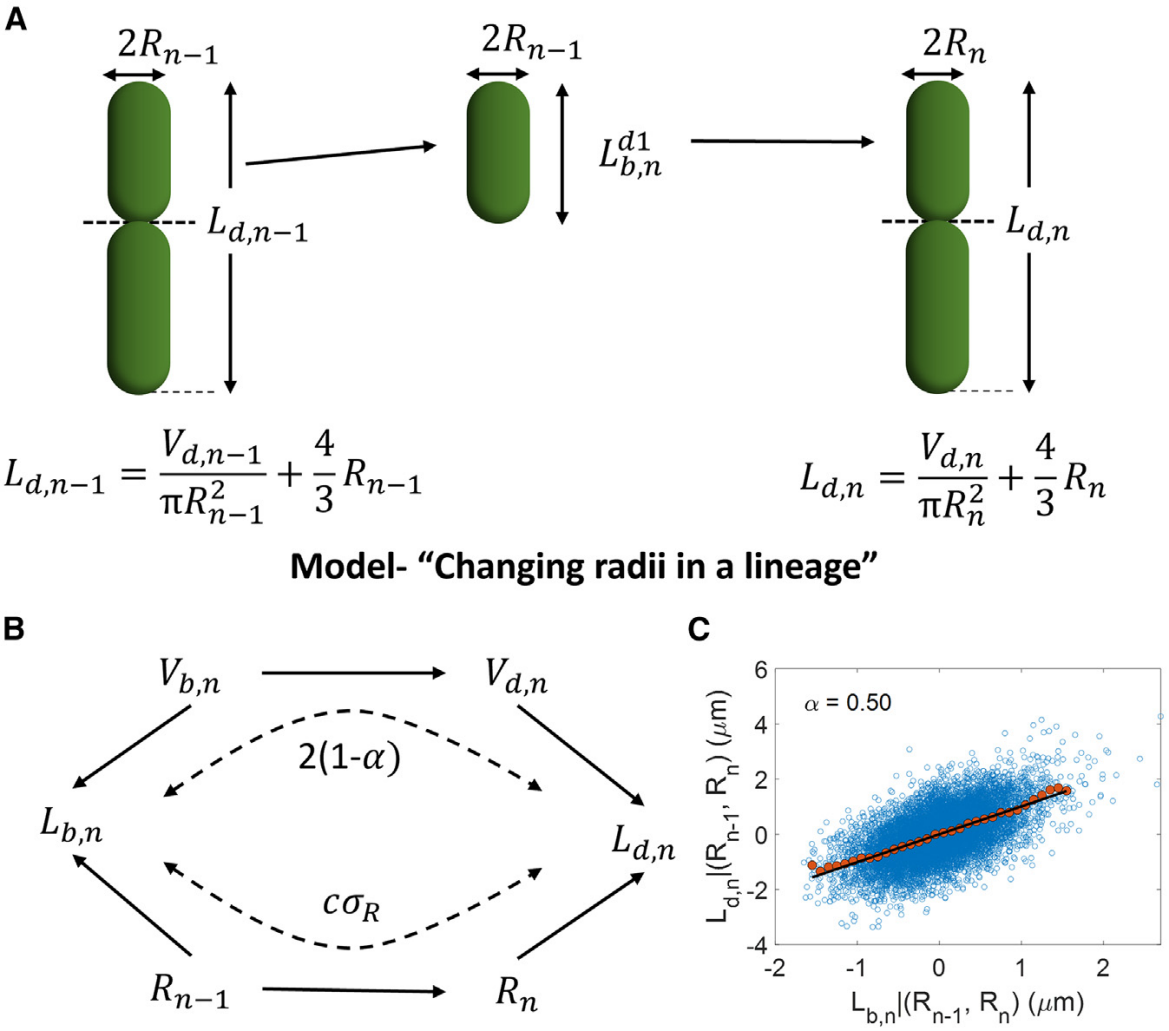
Model “changing radii in a lineage.” (*A*) Schematic of the model. The model assumes that cells have a spherocylindrical geometry and the division size is based on their birth size. There are already two fully formed cells just before cell division. The cells divide symmetrically by volume on average. The cell radii are correlated but not the same for successive generations. (*B*) Causal diagram showing that length at birth and division are correlated via two paths. One contribution is from the correlated radii in successive generations, and the other is from the division volume regulation strategy. The arrows in the graph point from cause to effect. (*C*) Residuals $L_{d,n}|\left(R_{n-1},R_{n}\right)$ versus $L_{b,n}|\left(R_{n-1},R_{n}\right)$ are plotted for simulations of the model in Fig. 3*A* with α=0.5. Using the slope of the best linear fit of the plot, we obtain an α (*top left*) consistent with the α used in the simulations.

The model discussed here (Fig. 3 *A*) varies in two aspects from the model discussed in the previous section. First, we consider a general model of cell division where the division volume $V_{d}$ is determined via the regulation strategy $f\left(V_{b}\right)$ discussed previously. Further, we relax the assumption that the cell width is fixed for a few generations in a lineage. During the cell cycle, there could be changes to the cell width such that the radius just before the cell division in generation n is

(2) $$R_{n}=R_{0}+c\left(R_{n-1}-R_{0}\right)+\sqrt{1-c^{2}}R_{0}\zeta_{R,n}\text{.}$$

The radius is correlated with that in the previous generation $\left(R_{n-1}\right)$, with c being the Pearson correlation coefficient of radii between generations. $\zeta_{R}$ is the noise in the radius assumed to be Gaussian with mean 0 and standard deviation $\sigma_{R}$. We assume that the radius does not change during the cell separation after the completion of septum formation. Thus, there are no sudden changes in cell lengths after the cell division process. The model also incorporates the spherocylinder cellular geometry and the two fully formed daughter cells at division features from the “pole formation before division” model.

Similar to the previous section, we can calculate the correlation between birth lengths k generations apart,

(3) $$r\left(k\right)=\frac{{\left(1-\alpha \right)}^{k}\frac{\alpha^{2}\sigma_{bd}^{2}+4\sigma_{m}^{2}}{\alpha \left(2-\alpha \right)}\frac{V_{0}^{2}}{{\left(\pi R_{0}^{2}\right)}^{2}}+4c^{k}{\left(\frac{R_{0}}{3}-\frac{V_{0}}{\pi R_{0}^{2}}\right)}^{2}\sigma_{R}^{2}}{\frac{\alpha^{2}\sigma_{bd}^{2}+4\sigma_{m}^{2}}{\alpha \left(2-\alpha \right)}\frac{V_{0}^{2}}{{\left(\pi R_{0}^{2}\right)}^{2}}+4{\left(\frac{R_{0}}{3}-\frac{V_{0}}{\pi R_{0}^{2}}\right)}^{2}\sigma_{R}^{2}}\text{.}$$

The length at birth in generation n+1 (or, equivalently, the length at division in generation n) is related to the birth length in generation n through two paths (Fig. 3 *B*): 1) via the cell cycle regulation strategy and 2) through the correlated cell radius across generations. The regulation strategy $f\left(V_{b}\right)$ leads to a correlation contribution of ${\left(1-\alpha \right)}^{k}\frac{\alpha^{2}\sigma_{bd}^{2}+4\sigma_{m}^{2}}{\alpha \left(2-\alpha \right)}\frac{V_{0}^{2}}{{\left(\pi R_{0}^{2}\right)}^{2}}$ between birth volumes k generations apart (first term in Eq. 3). The $4c^{k}{\left(\frac{R_{0}}{3}-\frac{V_{0}}{\pi R_{0}^{2}}\right)}^{2}\sigma_{R}^{2}$ term in Eq. 3 is the contribution of correlated radii across generations to the birth length correlations. We validate Eq. 3 using simulations of the “changing radii in a lineage” model. The correlations between birth lengths k generations apart were consistent between simulations and theory in Eq. 3 (Fig. S4 *A*). Further, theory and simulation results for the correlation between birth lengths were consistent when varying the cell width variability, $\sigma_{R}$ (Fig. S4 *B*). If $\sigma_{R}^{2}\ll \frac{\alpha^{2}\sigma_{bd}^{2}+4\sigma_{m}^{2}}{\alpha \left(2-\alpha \right)}$, then we recover the ${\left(1-\alpha \right)}^{k}$ decay of correlations with increasing k, consistent with a regulation strategy $f\left(V_{b}\right)$ and neglecting radius fluctuations (Fig. S4 *B*).

### Determining the volume cell cycle regulation strategy

We calculated the expression for the correlation between birth lengths k generations apart based on a general model of cell division, which accounts for cell width fluctuations. The cell width fluctuations confound the length correlations, thus hiding the value of the cell cycle regulation parameter, α. In this section, we discuss a method based on conditional correlations to estimate the value of α provided that the length at birth, the division length, and the cell radius for mother and daughter cells are measured.

A recent study used conditional correlations to restrict the cell cycle model space (15). The method entails calculating the correlation between two variables upon fixing the value of the third variable(s). This is equivalent to finding how two variables are correlated given the effects of the third variable(s) are removed. Upon removing the contribution of cell radius to cell lengths, a correlation between the transformed cell lengths would solely arise from the cell regulation strategy path illustrated in Fig. 3 *B*. The length variables $L_{b,n}$ and $L_{d,n}$ are dependent on radius values $R_{n-1}$ and $R_{n}$, respectively, in the general model discussed previously (Fig. 3 *A*). The residuals obtained upon linear regression of length variables, $L_{b/d,n}$, on both radii $R_{n-1}$ and $R_{n}$ $\left(L_{b/d,n}|\left(R_{n-1},R_{n}\right)\right)$ denotes variables that have no contribution from the cell radius. The slope of the best linear fit of the $L_{d}|\left(R_{n-1},R_{n}\right)$ versus $L_{b}|\left(R_{n-1},R_{n}\right)$ plot is equal to 2(1−α) (supporting material section S1.2.2). We verified it using simulations where we fixed the value of α to be 0.5 (Fig. 3 *C*). The α estimate obtained using the conditional correlation has only small errors when measurement errors in cell width measurements and correlated measurement noise are included (supporting material section S1.2.2; Fig. S5). Previous studies show that *E. coli* lengths agree well with a log-normal distribution (7). Hence, we also verified that our results do not change when we assume the birth and division lengths to be distributed lognormally instead of normally. For exponentially growing cells, we can simulate it by assuming a time-additive noise distributed normally rather than a normally distributed size-additive noise. We show the distribution of division length from one such simulation in Fig. S6 *B*. We also assume that cell width cannot be larger than a certain value (1.1 μm), mimicking the physical restrictions placed by the channels in mother machines. The cell radius distribution in the simulations is shown in Fig. S6 *C*. We find that the conditional correlations that we use to estimate α are independent of the nature of length and radius distributions (Fig. S6 *A*), and this holds over a range of cell width measurement noise.

### Test on experimental data

**Figure 4:**
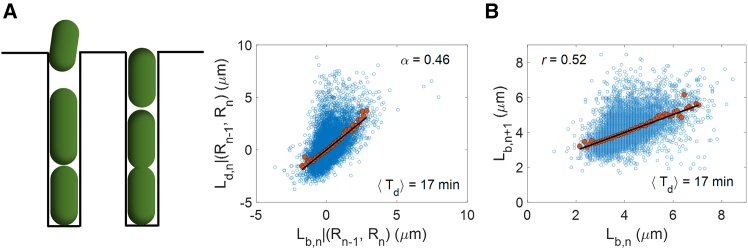
(*A*) Schematic diagram of a mother machine (*left*). Residuals $L_{d,n}|\left(R_{n-1},R_{n}\right)$ versus $L_{b,n}|\left(R_{n-1},R_{n}\right)$ are plotted (*right*) for *E. coli* experimental data obtained using mother machine experiments in (6) ($\left\langle T_{d}\right\rangle =$ 17 min, *n* = 17,055). The α value calculated is noted in the top right. (*B*) The $L_{b,n+1}$ versus $L_{b,n}$ plot is made for the same dataset in (*A*) (see supporting material section S3.1 for details about data analysis).

Next, we used the conditional correlation method on experimental datasets of *E. coli* collected in (6). Using microfluidic devices called mother machines, the study measures the birth lengths, division lengths, and cell radii for multiple single cells. Upon carrying out the conditional correlation analysis, we find the correlation to be close to 0.5 (Table 1). We show the plot for one of the growth conditions in Fig. 4 *A*. Thus, the α≈ 0.5 observed from experimental data implies that the underlying cell size regulation strategy cannot be a volume sizer. Similar estimates of the conditional correlation $\left(L_{b,n},L_{d,n}\right)|\left(R_{n-1},R_{n}\right)$ were obtained using *E. coli* data in other studies (Tables S1 (16) and S2 (9)).

**Table 1:** Pearson correlation coefficients along with their 95% confidence intervals are shown for *E. coli* cells growing in different growth media with mean generation times, $\left\langle T_{d}\right\rangle$

$\left\langle T_{d}\right\rangle$ (min)	No. of cells	$\left(L_{b,n},L_{d,n}\right)$	$\left(L_{b,n},L_{d,n}\right)|\left(R_{n-1}.R_{n}\right)$	$r\left(1\right);\left(L_{b,n},L_{b,n+1}\right)$
17	17,055	0.55 (0.54,0.56)	0.57 (0.56,0.58)	0.52 (0.51,0.53)
22	16,636	0.49 (0.48,0.50)	0.49 (0.48,0.51)	0.49 (0.48,0.50)
27	17,682	0.56 (0.55,0.57)	0.56 (0.55,0.57)	0.53 (0.52,0.54)
31	28,808	0.53 (0.52,0.54)	0.54 (0.53,0.54)	0.53 (0.52,0.53)
39	20,300	0.56 (0.55,0.56)	0.55 (0.55,0.56)	0.56 (0.55,0.57)
51	9763	0.55 (0.54,0.57)	0.54 (0.52,0.55)	0.59 (0.57,0.60)

The correlation between $L_{b}$ and $L_{d}$, $L_{b,n}$ and $L_{b,n+1}$, i.e., r(1), and conditional correlation, $\left(L_{b,n},L_{d,n}\right)|\left(R_{n-1},R_{n}\right)$, are shown for *E. coli* cells growing in six different growth media in (6). The 95% CIs are calculated as prescribed in supporting material section S2.1. CI, confidence interval.

We also analyzed wild-type fission yeast data (10) using conditional correlation $r\left(L_{b},L_{d}|R\right)$. We found the results to be consistent with the results in (10), which used a different method to estimate conditional correlation (supporting material section S5). However, to verify that fission yeast is indeed a sizer, more precise measurements of cell widths are needed. Furthermore, we used the method prescribed in (10) to analyze *E. coli* data. We found the results to be consistent with an adder model (supporting material section S5).

### Implications on cell width fluctuations

We can use the “changing radii in a lineage” model to predict the correlation between birth lengths in successive generations (k = 1 in Eq. 3). The values of the model parameters required to calculate the correlation using the model can be obtained from experimental data (see supporting material section S2.2 for the procedure and Table S3 for the values). Upon substituting these experimentally determined parameter values in Eq. 3, we expect the correlation between birth lengths in consecutive generations to be 0.62–0.82 (Table S3). This is inconsistent with the experimental results (Table 1; Fig. 4 *B*), where the correlations are close to 0.5 for all growth conditions studied.

These estimates for correlation are based on the assumption that there is negligible measurement noise. However, the length and width measurements will have variability contributions from the intrinsic stochasticity of the biochemical reactions as well as from inaccuracies in length measurements. Cell radii, which are of the order of 200–500 nm, will have significant measurement errors owing to their small magnitudes. Thus, the values of c and $\sigma_{R}$ that are substituted into Eq. 3 will be imprecise. Next, we try to estimate the values of c and $\sigma_{R}$ that might reconcile α = 0.5 estimated previously with correlation = 0.5 from experiments.

We simplify Eq. 3 by assuming that the length of the cell is generally larger than the cell radius, i.e., $\frac{V_{0}}{\pi R_{0}^{2}}>\frac{R_{0}}{3}$. Thus, the correlation between birth lengths in consecutive generations is approximately only a function of noise variables and c. Substituting α = 0.5 in Eq. 3,

(4) $$r\left(1\right)\approx \frac{\frac{1}{2}\frac{\sigma_{bd}^{2}+16\sigma_{m}^{2}}{3}+4c\sigma_{R}^{2}}{\frac{\sigma_{bd}^{2}+16\sigma_{m}^{2}}{3}+4\sigma_{R}^{2}}\text{.}$$

We find that r(1) will be 0.5 in two cases—either c = 0.5 or $\sigma_{R}\ll \sqrt{\sigma_{bd}^{2}+16\sigma_{m}^{2}}$. Although either of these cases is possible in principle, strategies to regulate the radius across generations to be 0.5 are unknown. Thus, we conclude that the intrinsic variability of radius $\left(\sigma_{r}\right)$ is much smaller than the noise in setting the division length. Most of the measured cell width variability has large contributions from measurement errors.

Until now, we have focused on models where cell volume is the relevant quantity the cell controls. The underlying assumption is that the rate of volume growth is proportional to the rate of protein synthesis and biomass accumulation. (17) observed that the cell surface area/biomass ratio remains constant during the cell cycle while density changes. This would mean that cell surface area, instead of the cell volume, is the appropriate proxy for biomass accumulation. However, our conclusions about the actual cell width variability being small remain unchanged even if we consider the surface area being controlled (supporting material section S4).

A striking observation in the experimental data is that the correlations between $L_{b,n}$ and $L_{d,n}$ closely agree with the conditional correlations $\left(L_{b,n},L_{d,n}\right)|\left(R_{n-1},R_{n}\right)$ (Tables 1, S1, and S2). Next, we use this observation and cell cycle model simulations to restrict the values of $\sigma_{R}$ and c. In the simulations, we allow for a difference of 0.02 between the two correlation values. Most of the correlation differences obtained from experiments are within this limit (Tables 1, S1, and S2). We vary $\sigma_{R}$ and c while keeping the measured values of radius CV $\left(\sigma_{Rt}\right)$ and correlation between consecutive generations $\left(c_{t}\right)$ fixed. We restrict the parameter space in the simulations such that the correlation between measurement noise in the radius in successive generations is between 0 and 1. For the division regulation strategy $f\left(V_{b}\right)$, we find that the correlation and conditional correlation are close for smaller values of $\sigma_{R}$ (Fig. S7 *A*). We find that the measurement noise in width accounts for at least half of the total width variability. Similar results are obtained for cell cycle models beyond the $f\left(V_{b}\right)$ regulation strategy (Fig. S7, *B*–*D*).

Thus, the correlation and conditional correlation values bound the intrinsic cell width variability to be, at most, half the width variability observed in experiments.

## Conclusion

Our previous work has shown that unaccounted sources of noise could lead to misinterpretation of the results of single-cell data analysis methods (18). (10) presented another example of the misinterpretation where width fluctuations would lead to a volume sizer being misinterpreted as a length adder (Figs. 1 *B* and 2, *A* and *B*). In a volume sizer, division happens when a certain sizer protein reaches a threshold amount irrespective of its initial amount at birth, and the protein biosynthesis is coupled to volume growth. Note that the underlying mechanism might not involve sensing the absolute protein numbers in the cell. The sizer proteins can be limited to a region of particular size. The rise in their concentration (or equivalently number) in this fixed region to a threshold amount can trigger division, as is found in fission yeast (19). In contrast, for a length adder, division happens when the protein accumulates a critical amount from birth and the protein biosynthesis is coupled to length growth. Thus, two differences arise between these two models: 1) what triggers division, is it the protein accumulation by a threshold amount or the protein number reaches a critical amount? 2) is the biomass accumulation related to length or volume?

Cell volume is also a suitable candidate to be the proxy for cell size. Biochemical reaction kinetics in the cell depend on the concentration of reactant species, which is tied to the cell volume. (20) also showed that the average cell volume, rather than the cell length, surface area, and width, scaled as $e^{\left\langle \lambda \right\rangle \left(C+D\right)}$, where ⟨λ⟩ is the average growth rate and C+D is the average time between the start of DNA replication and division. This observation can be explained by assuming that the average volume per origin of replication at the initiation of DNA replication is a constant, and division happens after a constant C+D time from birth (21). In fast and intermediate growth conditions, the C+D time was found to be 60 min (22). Since cell volume is the only cell geometry characteristic that follows the growth law, it is natural to assume that cell volume per origin is regulated at the start of DNA replication (23). Thus, assuming a volumetric control, we find from Eq. 4 that length and volume correlations can be the same if intrinsic radius variability is small compared to other fluctuations (division size or division ratio). Note that for a spherocylindrical cell geometry with negligible cell width variability, volume at birth or division is a linear function and not proportional to the cell length. However, cell cycle analysis frequently uses Pearson correlation coefficients, which are invariant under linear transformations. Hence, it is often equivalent to use volume or length in the cell cycle data analysis.

The mechanistic understanding of width control in bacteria is still an open question of research (24,25,26,27). Our results suggest that width control in *E. coli* is even more accurate than previously thought. We found that length correlations between birth and division and the conditional correlation between them when conditioned upon the radius in the mother cell and the current generation were extremely close in experimental data in multiple growth conditions from multiple studies (Tables 1, S1, and S2) (6,9,16). Using simulations of cell cycle models (Fig. S7), we found the range of intrinsic radius variability for which the above equality in correlations hold. The simulations indicate that the variance contribution of the measurement noise in the radius can be at least half of the total radius variability. If the CV of the radius is approximately 6% (Table S3), then our estimates put the CV of the intrinsic radius variability to be less than 4% (standard deviation ≈ 10 nm). In general, we conclude that the measured cell width variability is dominated by measurement noise. Hence, more accurate measurements, possibly using electron microscopy, are required to measure the actual (intrinsic) radius variability.

More broadly, adder behavior has been observed in multiple species across different domains of life (2,7,28,29,30). For a volume sizer to appear as a length adder requires fine-tuning cell cycle parameters, such as cell radius variability and noise in setting the division size. It is unlikely that the parameters are precisely controlled in these different organisms with different geometries and underlying cell cycle mechanisms. Why the adder behavior is so ubiquitous remains an open question.

## Acknowledgments

We thank Martin Howard, Sven van Teeffelen, and Sattar Taheri-Araghi for the useful feedback on the manuscript. A.A. and P.K. acknowledge support from NSF CAREER 1752024. A.A. is thankful for the generous support from the Clore Center for Biological Physics and ERC-CoG 2023 101125981.

## Author contributions

A.A. and P.K. conceptualized the project, P.K. carried out the analysis, and P.K. and A.A. wrote the draft and reviewed and edited the manuscript. A.A. acquired funding.

## Declaration of interests

The authors declare no competing interests.

## Supporting material

Document S1. Figures S1–S9, Tables S1–S3, and supporting material

Document S2. Article plus supporting material
