# Supplementary material for: Are cell length and volume interchangeable in cell cycle analysis?
Source: Biophys J. 2025 Mar 26;124(9):1424–32. doi: 10.1016/j.bpj.2025.03.019 (PMC12256882; doi:10.1016/j.bpj.2025.03.019)
Supplement: Document S2. Article plus supporting material [file mmc2.pdf]

Biophysical Journal
Article

Biophysical Society

Are cell length and volume interchangeable in cell cycle analysis?

Prathitha Kar[1,2] and Ariel Amir[3,*]
[1]Department of Chemistry and Chemical Biology, Harvard University, Cambridge, Massachusetts; [2]School of Engineering and Applied Sciences, Harvard University, Cambridge, Massachusetts; and [3]Department of Physics of Complex Systems, Weizmann Institute of Science, Rehovot, Israel

ABSTRACT Cell length has been used as a proxy for cell size in cell cycle modeling studies. A previous study, however, brought into question the validity of this assumption, noting that correlations between cell lengths can be different from those involving cell volume if cell width fluctuations are taken into account. If cell volume is regulated, data analysis involving cell lengths will lead to an incorrect inference of the cell size control mechanism. We used conditional correlation of length variables conditioned upon radius variables to elucidate the underlying volume control mechanism. Using the conditional correlation on previous mother machine datasets measuring lengths at birth and division and the cell radius for multiple cells, we find that the cell volume control strategy is consistent with an adder model. Further, using the conditional correlation, we conclude that measurement noise constitutes a significant portion of the radius variability in the experimental datasets. To conclude, cell length and cell volume can often be used interchangeably owing to small cell width fluctuations.

SIGNIFICANCE Cell cycle studies regularly use correlations between cell lengths at various cell cycle checkpoints to elucidate the underlying mechanisms. It is unclear whether length is a good proxy for cell volume. Indeed, studies in fission yeast showed that cell width fluctuations can influence the measured correlations and make length and volume noninterchangeable. Using modeling and data analysis of *Escherichia coli* experimental data, we find that cell width fluctuations have a negligible impact on the correlation structure of cell cycle variables. This implies that for bacteria such as *E. coli*, length and volume can often be used interchangeably. Our analysis suggests that cell width is tightly regulated in *E. coli* to an accuracy of ≈4% or 10 nm.

INTRODUCTION

Recent developments in microscopy and microfluidics have enabled researchers to study cell cycle regulation at a single-cell level (1). Data analysis methods and quantitative models complement these experiments, allowing us to make progress in our understanding of cell size homeostasis. Coarse-grained phenomenological models agnostic of the molecular details have provided clues about cell size regulation mechanisms (2–4). Often, the Pearson correlation coefficient and best linear fit predictions from models are compared against experimental data to uncover the underlying biological mechanism. We show an example (Fig. 1 *A*) where the adder model of cell division—cells divide upon adding a constant size from birth (*black dashed line*)—is consistent with the binned data (*red points*) from experiments on *Escherichia coli* (5–9). Throughout this paper, binned data are calculated by dividing the x axis values into equal-width bins and finding the mean of the y axis values. In this analysis, cell length is assumed to be a proxy for cell volume, and they are often used interchangeably.

(10) argued that they are not interchangeable cell characteristics. Their logic followed a scenario pointed out in (2). (10) discussed that subpopulations with different mean lengths at birth and division can arise despite carefully maintained growth conditions in microfluidics experiments due to natural cell width variability. For a population of cells with multiple subpopulations differing in their average length at birth and division, a division strategy called the sizer model, where cells divide upon reaching a critical size (slope = 0 in the L_d versus L_b plot in each subpopulation) can be misconstrued as an adder (slope = 1 in the L_d versus L_b plot) when the whole population is considered (Fig. 1 *B*). This is the so-called "Simpson's paradox" and,

Submitted June 1, 2024, and accepted for publication March 21, 2025.
*Correspondence: ariel.amir@weizmann.ac.il
Editor: Jennifer Schwarz.
https://doi.org/10.1016/j.bpj.2025.03.019
© 2025 Published by Elsevier Inc. on behalf of Biophysical Society.
This is an open access article under the CC BY license (http://creativecommons.org/licenses/by/4.0/).

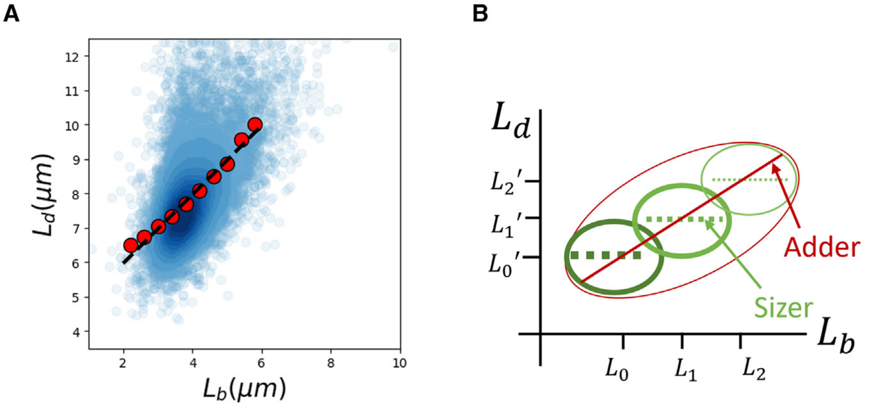

FIGURE 1 (*A*) An example where the adder model of cell division, in which cells divide upon adding a constant size from birth, is consistent with the experimental data of *E. coli*. The blue cloud is the raw data obtained from (6) growing in a growth medium with the average doubling time ($\langle T_d \rangle$) = 17 min (no. of cells n = 18,248). The red dots are the experimental binned data trend calculated by dividing the x axis values into bins of equal width and calculating the mean of the y axis values in that bin. The binned data trend matches the prediction for the binned data trend of the adder model (*black dashed line* with slope = 1). (*B*) Illustration of Simpson's paradox affecting length correlations. The underlying model could be a volume sizer with width fluctuations. Each green cloud corresponds to cells of different radii and is a volume sizer. Each subpopulation with a different radius is also a length sizer but with different average birth and division lengths. When the population of cells of different radii is combined, the correlation resembles that of a length adder.

in this case, leads to the actual sizer model being interpreted as an adder when various subpopulations are analyzed together. They found that cell width fluctuations could explain the positive length correlations observed in experimental data on fission yeast despite each subpopulation being a sizer. Using the same argument for *E. coli*, they found that a volume sizer, along with cell width fluctuations, could lead to a slope = 1 for the best linear fit of the L_d versus L_b plot. The underlying biological mechanisms leading to a sizer differ from those resulting in an adder. This would mean that cell cycle analyses involving cell length data require width fluctuations to be accounted for. An important thing to note is that the measured cell radius variability is small ($\approx 6\%$) (6). However, calculations in (10) and this paper show that the slope of L_d versus L_b is dependent on the ratio of radius variability and the noise in setting the division volume. Since the division volume noise is also small, there could be a substantial difference between the slope calculated using the entire population of cells and each subpopulation, even for a small radius variability.

In this paper, we discuss various cell division models that take cell width fluctuations into account. First, we show that the model proposed in (10) is inconsistent with experimental data from *E. coli* regarding the correlation between birth lengths in successive generations. Based on a general model of cell division and cell width fluctuations, we devise a method involving conditional correlation of length variables conditioned upon radius variables to elucidate the underlying division volume control mechanism. Upon accounting for the width fluctuations, we find *E. coli* experimental data to be consistent with an adder model of cell division. Using conditional correlations, we also estimate that the actual radius variability is, at most, half of the measured variability, indicating that measurement noise is a major contributor to the radius measurements. This supports the assumptions from previous studies that cell lengths and volumes can be used interchangeably.

RESULTS AND DISCUSSION

First, we will state the model proposed in (10) and obtain the correlation structure based on it. Using the model, we will restate the results in (10) and Fig. 1 *B* related to the correlation between the length at birth and division. Next, we will show that the minimal model fails to recover the correlations between birth lengths in successive generations.

In the model, *E. coli* single-cell geometry is approximated as a spherocylinder with identical cell radii (R) within a cell lineage (Fig. 2 *A*). However, in a typical cell cycle analysis such as the one in Fig. 1 *A*, the data combine cells from different lineages, which might have different cell widths (and equivalently radii). One scenario where such cell radius variation might arise is if the cell width varies at a timescale much larger than the doubling time of cells. In the subsequent section, we will discuss a model where the cell radius changes in consecutive generations of a cell lineage. We assume that the cell divides when it reaches a critical volume $2\,V_0$ (volume sizer). This is a special case of the general model where the volume at birth determines the cell division volume via a regulation strategy, $f(V_b)$ (11). A simple choice of $f(V_b)$ is $f(V_b) = 2(1-\alpha)V_b + 2\alpha V_0$, where α is the cell cycle regulation parameter. Mathematically, we can express the division volume to be $V_d = 2(1-\alpha)V_b + 2\alpha V_0(1+\zeta_s)$, where ζ_s is the size additive division noise. α can assume any value between 0 and 2, with $\alpha = \frac{1}{2}$ being the adder strategy and $\alpha = 1$ the sizer strategy. The cells divide symmetrically on average, i.e., the mean division ratio $= \frac{1}{2}$ and the noise in division ratio for cells dividing in generation $n = \delta_n$. Upon dividing, the two daughter cells develop hemispherical poles at one end, keeping the total volume constant, i.e., the division volume of the mother cell is equal to the sum of the birth volumes of the two daughter cells. This amounts to an addition of $\frac{R}{3}$ term to the birth lengths ("pole formation after division" model in Fig. 2 *A*). (10)

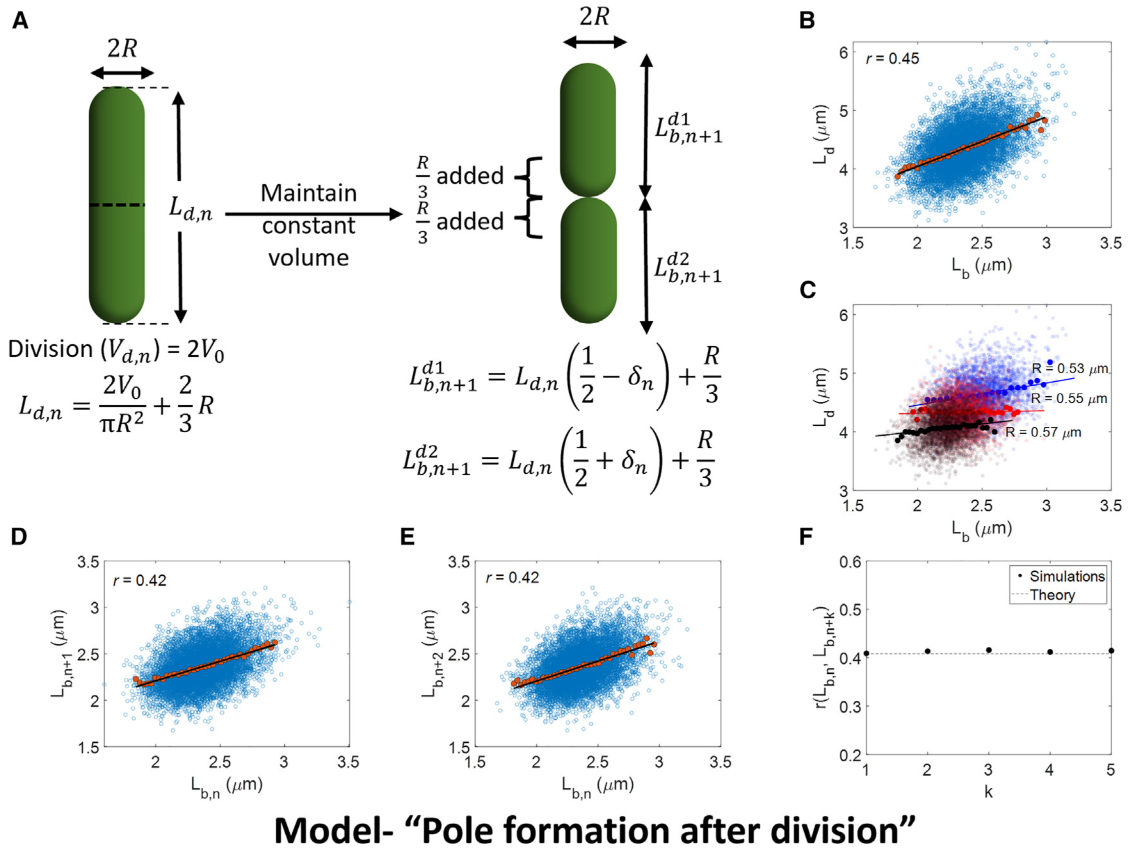

FIGURE 2 Model “pole formation after division.” (*A*) Schematic of the model proposed in (10). The cell divides when it reaches a critical volume 2 V_0 (volume sizer). The cells divide symmetrically by length on average, i.e., the mean division ratio $= \frac{1}{2}$ and the noise in division ratio for cells dividing in generation $n = \delta_n$. Upon dividing, the two daughter cells develop hemispherical poles at one end, rapidly keeping the total volume constant, i.e., the division volume of the mother cell is equal to the sum of the birth volumes of the two daughter cells. This amounts to an addition of $\frac{R}{3}$ term to the birth lengths. The cell radius is fixed for all cells in a particular lineage but varies for different lineages. (*B–E*) Simulations of the model in (*A*) are carried out for 10,000 cell lineages and over 25 generations. For the simulations, (*B*) we plot the L_d versus L_b plot. The correlation r (*top left*) points to a near-adder model. (*C*) We plot L_d versus L_b for small subsets of R. We arrange the simulated dataset in ascending order of radius and divide it into three groups. For each group (with a different average radius), we plot the L_d versus L_b plot. The plot shows the Simpson’s paradox mentioned in Fig. 1 *B*. (*D*) Length at birth in generation $n+1$ versus generation n is plotted. (*E*) Length at birth in generation $n+2$ versus generation n is plotted. The correlation values are identical and consistent with Eq. 1. In all plots, the cloud is the raw data, the dots represent the binned data, and the line is the best linear fit. As is the case throughout the paper, binned data are calculated by dividing the x axis values into equal-width bins and taking the mean of the y axis values in those bins. (*F*) We calculate the correlation between the lengths at birth in generation n and k generations later for different k. Consistent with Eq. 1 and (*D*) and (*E*), we find the correlations to be constant independent of k.

based the model on fission yeast growth, where a rapid increase in cell lengths is observed just after cell birth (12). A similar new pole formation pattern is observed in bacterial species such as *Bacillus subtilis* (13). In the next section, we will discuss a model of pole formation found in *E. coli* where cells start constricting and form the new hemispherical poles before division.

Upon simulating the model with the same model parameters as in (10), we recover their result of slope ≈ 1 for the best linear fit of the L_d versus L_b plot, which is consistent with a length adder (Fig. 2 *B*; supporting material section S1.1.1). As stated previously, this positive correlation comes from Simpson’s paradox (Fig. 1 *B*): consider cells with radii δR larger than the average. Such cells would have an average length at birth and division different from that of cells with radii δR smaller than average. Within each subpopulation, cells divide upon reaching a critical volume or equivalently critical length, as the cell width within the subpopulation is fixed (Fig. 2 *C*). Upon combining the different subpopulations, the shifts due to different length averages lead to an apparent length adder (Fig. 2 *B*).

Minimal cell division models that neglect width fluctuations are consistent with the experimental data regarding the correlation between cell birth lengths in successive generations. According to this model, the birth lengths n generations apart are correlated as $(1-\alpha)^n$ (4,11). (4) analyzed experimental data on *E. coli* from (14) and found the Pearson correlation coefficient between the birth lengths of mother and daughter cells to be approximately 0.5. The correlation between the birth lengths of mother and

granddaughter cells was approximately 0.25. Together, these correlations point to $\alpha = 0.5$, i.e., the adder model. We tested whether the model that includes width fluctuations also agrees with these correlations. Using the model, we found the correlation between length at birth at generation n ($L_{b,n}$) and k generations later ($L_{b,n+k}$) (supporting material section S1.1.2) to be

$$r = \frac{4\left(\frac{R_0}{3} - \frac{V_0}{\pi R_0^2}\right)^2 \sigma_R^2}{\left(\frac{V_0}{\pi R_0^2}\right)^2 \sigma_{bd}^2 + 4\left(\frac{R_0}{3} - \frac{V_0}{\pi R_0^2}\right)^2 \sigma_R^2 + \left(\frac{V_0}{\pi R_0^2} + \frac{R_0}{3}\right)^2 \sigma_m^2}, \tag{1}$$

where R_0 is the mean radius, σ_{bd} is the standard deviation of division noise ζ_s, σ_R is the coefficient of variation (CV) in radius, and σ_m is the standard deviation of division ratio δ_n. Contrary to the decreasing correlations with increasing k that have been observed for experimental data (4), we found that correlations between successive birth lengths are constant and independent of k for this model (Eq. 1; Fig. 2, *D–F*). In this model, cell lengths have no memory of the prior generations (sizer) except for having the same radius. Since the radius stays constant within a lineage independent of k, the correlations do not vary despite considering cell lengths k generations apart. This is evident in Eq. 1, where the covariance between birth lengths k generations apart (the numerator in Eq. 1) is only dependent on the variability in cell width. The positive correlations in this case have the same origin as before, i.e., Simpson's paradox (Fig. S1).

Thus, our analysis shows that a volume sizer with width fluctuations cannot account for the correlations between birth lengths observed in experimental data.

Correlation structure in a cell division model where the new pole forms at mid-cell before division

In the previous model, hemispherical pole formation followed the cell division event. We wanted to verify that the timing of the new pole formation does not affect the correlation structure obtained from the model. The model in the previous section assumed a sudden increase in cell length in a small period around birth, which is not observed in *E. coli*. Therefore, we test a model of cell growth and division where cells constrict and form new poles before dividing ("pole formation before division" model).

The model borrows characteristics from the previous one, such as spherocylindrical cell geometry, constant radius along a lineage while variability exists across lineages, and a volume sizer. However, in this model, we assume that two spherocylindrical daughter cells are fully formed just before division. The two daughter cells are symmetrical on average, but there are fluctuations in the volume division ratio denoted by δ_n. For an asymmetrical division in generation n, it is assumed that one of the cells receives an additional $\delta_n V_{d,n}$ in volume. However, the length partition for that cell is not $\delta_n L_{d,n}$ since cell length is a linear function of volume (Fig. S2 *A*). Partitioning by length does not impact the results qualitatively (Fig. S3, *A–C*).

We find that, for the same parameters as in the previous section, the Pearson correlation coefficient between L_b and L_d is close to 0.5, thus agreeing with a length adder (Fig. S2 *B*). It follows the same explanation as the previous section, where Simpson's paradox leads to an apparent adder (Fig. S2 *C*). Importantly, the correlation between birth lengths, k generations apart, is a constant for $k = 1$ and 2 (Fig. S2, *D* and *E*; supporting material section S1.2). As was the case previously, the constancy in correlation k generations apart is because the lengths are correlated owing to the same radius in a lineage. Thus, the model fails in manner similar to that of the previous model.

Correlation structure in a model where radius correlations between generations are not equal to one

Both previous models show that the volume sizer can lead to a length adder, but they do not agree with all the correlations observed in the experiments. The correlations between length births across generations do not decay because the radius in a lineage is fixed. However, if the cell width fluctuates on a timescale of cell doubling time, then the radius might be correlated but not equal to one between successive generations. In this section, we will discuss a model where we relax the assumption that the radius is fixed in a lineage ("changing radii in a lineage" model).

The model discussed here (Fig. 3 *A*) varies in two aspects from the model discussed in the previous section. First, we consider a general model of cell division where the division volume V_d is determined via the regulation strategy $f(V_b)$ discussed previously. Further, we relax the assumption that the cell width is fixed for a few generations in a lineage. During the cell cycle, there could be changes to the cell width such that the radius just before the cell division in generation n is

$$R_n = R_0 + c(R_{n-1} - R_0) + \sqrt{1 - c^2} R_0 \zeta_{R,n}. \tag{2}$$

The radius is correlated with that in the previous generation (R_{n-1}), with c being the Pearson correlation coefficient of radii between generations. ζ_R is the noise in the radius assumed to be Gaussian with mean 0 and standard deviation σ_R. We assume that the radius does not change during the cell separation after the completion of septum formation. Thus, there are no sudden changes in cell lengths after the cell division process. The model also incorporates the spherocylinder cellular geometry and the two fully formed

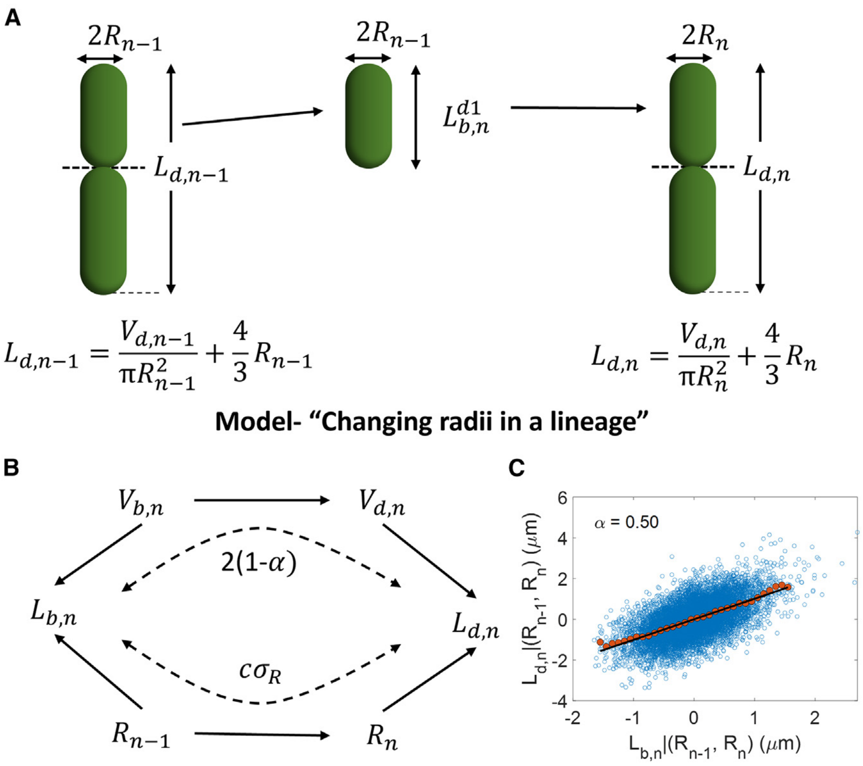

FIGURE 3 Model "changing radii in a lineage." (*A*) Schematic of the model. The model assumes that cells have a spherocylindrical geometry and the division size is based on their birth size. There are already two fully formed cells just before cell division. The cells divide symmetrically by volume on average. The cell radii are correlated but not the same for successive generations. (*B*) Causal diagram showing that length at birth and division are correlated via two paths. One contribution is from the correlated radii in successive generations, and the other is from the division volume regulation strategy. The arrows in the graph point from cause to effect. (*C*) Residuals $L_{d,n}\left|(R_{n-1}, R_n)\right.$ versus $L_{b,n}\left|(R_{n-1}, R_n)\right.$ are plotted for simulations of the model in Fig. 3 *A* with $\alpha = 0.5$. Using the slope of the best linear fit of the plot, we obtain an α (*top left*) consistent with the α used in the simulations.

daughter cells at division features from the "pole formation before division" model.

Similar to the previous section, we can calculate the correlation between birth lengths k generations apart,

$$r(k) = \frac{(1-\alpha)^k \frac{\alpha^2\sigma_{bd}^2 + 4\sigma_m^2}{\alpha(2-\alpha)} \frac{V_0^2}{\left(\pi R_0^2\right)^2} + 4c^k\left(\frac{R_0}{3} - \frac{V_0}{\pi R_0^2}\right)^2 \sigma_R^2}{\frac{\alpha^2\sigma_{bd}^2 + 4\sigma_m^2}{\alpha(2-\alpha)} \frac{V_0^2}{\left(\pi R_0^2\right)^2} + 4\left(\frac{R_0}{3} - \frac{V_0}{\pi R_0^2}\right)^2 \sigma_R^2}. \quad (3)$$

The length at birth in generation $n+1$ (or, equivalently, the length at division in generation n) is related to the birth length in generation n through two paths (Fig. 3 *B*): 1) via the cell cycle regulation strategy and 2) through the correlated cell radius across generations. The regulation strategy $f(V_b)$ leads to a correlation contribution of $(1-\alpha)^k \frac{\alpha^2\sigma_{bd}^2+4\sigma_m^2}{\alpha(2-\alpha)} \frac{V_0^2}{\left(\pi R_0^2\right)^2}$ between birth volumes k generations apart (first term in Eq. 3). The $4c^k\left(\frac{R_0}{3} - \frac{V_0}{\pi R_0^2}\right)^2 \sigma_R^2$ term in Eq. 3 is the contribution of correlated radii across generations to the birth length correlations. We validate Eq. 3 using simulations of the "changing radii in a lineage" model. The correlations between birth lengths k generations apart were consistent between simulations and theory in Eq. 3 (Fig. S4 *A*). Further, theory and simulation results for the correlation between birth lengths were consistent when varying the cell width variability, σ_R (Fig. S4 *B*). If $\sigma_R^2 \ll \frac{\alpha^2\sigma_{bd}^2+4\sigma_m^2}{\alpha(2-\alpha)}$, then we recover the $(1-\alpha)^k$ decay of correlations with increasing k, consistent with a regulation strategy $f(V_b)$ and neglecting radius fluctuations (Fig. S4 *B*).

Determining the volume cell cycle regulation strategy

We calculated the expression for the correlation between birth lengths k generations apart based on a general model of cell division, which accounts for cell width fluctuations. The cell width fluctuations confound the length correlations, thus hiding the value of the cell cycle regulation parameter, α. In this section, we discuss a method based on conditional correlations to estimate the value of α provided that the

TABLE 1 Pearson correlation coefficients along with their 95% confidence intervals are shown for *E. coli* cells growing in different growth media with mean generation times, $\langle T_d \rangle$

$\langle T_d \rangle$ (min)	No. of cells	$(L_{b,n}, L_{d,n})$	$(L_{b,n}, L_{d,n}) \vert (R_{n-1}.R_n)$	$r(1); (L_{b,n}, L_{b,n+1})$
17	17,055	0.55 (0.54,0.56)	0.57 (0.56,0.58)	0.52 (0.51,0.53)
22	16,636	0.49 (0.48,0.50)	0.49 (0.48,0.51)	0.49 (0.48,0.50)
27	17,682	0.56 (0.55,0.57)	0.56 (0.55,0.57)	0.53 (0.52,0.54)
31	28,808	0.53 (0.52,0.54)	0.54 (0.53,0.54)	0.53 (0.52,0.53)
39	20,300	0.56 (0.55,0.56)	0.55 (0.55,0.56)	0.56 (0.55,0.57)
51	9763	0.55 (0.54,0.57)	0.54 (0.52,0.55)	0.59 (0.57,0.60)

The correlation between L_b and L_d, $L_{b,n}$ and $L_{b,n+1}$, i.e., $r(1)$, and conditional correlation, $(L_{b,n}, L_{d,n}) \vert (R_{n-1}, R_n)$, are shown for *E. coli* cells growing in six different growth media in (6). The 95% CIs are calculated as prescribed in supporting material section S2.1. CI, confidence interval.

length at birth, the division length, and the cell radius for mother and daughter cells are measured.

A recent study used conditional correlations to restrict the cell cycle model space (15). The method entails calculating the correlation between two variables upon fixing the value of the third variable(s). This is equivalent to finding how two variables are correlated given the effects of the third variable(s) are removed. Upon removing the contribution of cell radius to cell lengths, a correlation between the transformed cell lengths would solely arise from the cell regulation strategy path illustrated in Fig. 3 *B*. The length variables $L_{b,n}$ and $L_{d,n}$ are dependent on radius values R_{n-1} and R_n, respectively, in the general model discussed previously (Fig. 3 *A*). The residuals obtained upon linear regression of length variables, $L_{b/d,n}$, on both radii R_{n-1} and R_n ($L_{b/d,n} \vert (R_{n-1}, R_n)$) denotes variables that have no contribution from the cell radius. The slope of the best linear fit of the $L_d \vert (R_{n-1}, R_n)$ versus $L_b \vert (R_{n-1}, R_n)$ plot is equal to $2(1-\alpha)$ (supporting material section S1.2.2). We verified it using simulations where we fixed the value of α to be 0.5 (Fig. 3 *C*). The α estimate obtained using the conditional correlation has only small errors when measurement errors in cell width measurements and correlated measurement noise are included (supporting material section S1.2.2; Fig. S5). Previous studies show that *E. coli* lengths agree well with a log-normal distribution (7). Hence, we also verified that our results do not change when we assume the birth and division lengths to be distributed lognormally instead of normally. For exponentially growing cells, we can simulate it by assuming a time-additive noise distributed normally rather than a normally distributed size-additive noise. We show the distribution of division length from one such simulation in Fig. S6 *B*. We also assume that cell width cannot be larger than a certain value (1.1 μm), mimicking the physical restrictions placed by the channels in mother machines. The cell radius distribution in the simulations is shown in Fig. S6 *C*. We find that the conditional correlations that we use to estimate α are independent of the nature of length and radius distributions (Fig. S6 *A*), and this holds over a range of cell width measurement noise.

Test on experimental data

Next, we used the conditional correlation method on experimental datasets of *E. coli* collected in (6). Using microfluidic devices called mother machines, the study measures the birth lengths, division lengths, and cell radii for multiple single cells. Upon carrying out the conditional correlation analysis, we find the correlation to be close to 0.5 (Table 1). We show the plot for one of the growth conditions in Fig. 4 *A*. Thus, the $\alpha \approx$ 0.5 observed from experimental data implies that the underlying cell size regulation strategy cannot be a volume sizer. Similar estimates of the conditional correlation $(L_{b,n}, L_{d,n}) \vert (R_{n-1}, R_n)$ were obtained using *E. coli* data in other studies (Tables S1 (16) and S2 (9)).

We also analyzed wild-type fission yeast data (10) using conditional correlation $r(L_b, L_d \vert R)$. We found the results to be consistent with the results in (10), which used a different method to estimate conditional correlation (supporting material section S5). However, to verify that fission yeast is indeed a sizer, more precise measurements of cell widths are needed. Furthermore, we used the method prescribed

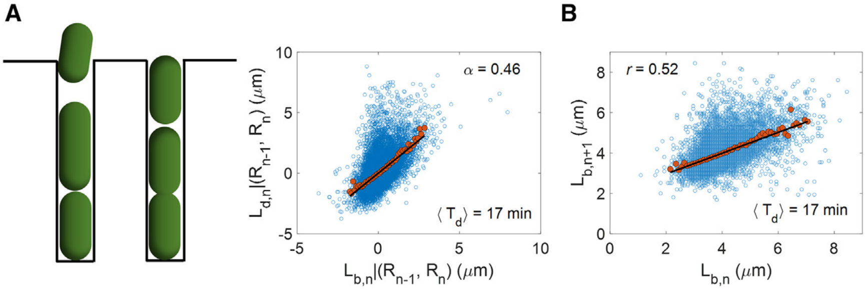

FIGURE 4 (*A*) Schematic diagram of a mother machine (*left*). Residuals $L_{d,n} \vert (R_{n-1}, R_n)$ versus $L_{b,n} \vert (R_{n-1}, R_n)$ are plotted (*right*) for *E. coli* experimental data obtained using mother machine experiments in (6) ($\langle T_d \rangle$ = 17 min, n = 17,055). The α value calculated is noted in the top right. (*B*) The $L_{b,n+1}$ versus $L_{b,n}$ plot is made for the same dataset in (*A*) (see supporting material section S3.1 for details about data analysis).

in (10) to analyze *E. coli* data. We found the results to be consistent with an adder model (supporting material section S5).

Implications on cell width fluctuations

We can use the "changing radii in a lineage" model to predict the correlation between birth lengths in successive generations ($k = 1$ in Eq. 3). The values of the model parameters required to calculate the correlation using the model can be obtained from experimental data (see supporting material section S2.2 for the procedure and Table S3 for the values). Upon substituting these experimentally determined parameter values in Eq. 3, we expect the correlation between birth lengths in consecutive generations to be 0.62–0.82 (Table S3). This is inconsistent with the experimental results (Table 1; Fig. 4 *B*), where the correlations are close to 0.5 for all growth conditions studied.

These estimates for correlation are based on the assumption that there is negligible measurement noise. However, the length and width measurements will have variability contributions from the intrinsic stochasticity of the biochemical reactions as well as from inaccuracies in length measurements. Cell radii, which are of the order of 200–500 nm, will have significant measurement errors owing to their small magnitudes. Thus, the values of c and σ_R that are substituted into Eq. 3 will be imprecise. Next, we try to estimate the values of c and σ_R that might reconcile $\alpha = 0.5$ estimated previously with correlation $= 0.5$ from experiments.

We simplify Eq. 3 by assuming that the length of the cell is generally larger than the cell radius, i.e., $\frac{V_0}{\pi R_0^2} > \frac{R_0}{3}$. Thus, the correlation between birth lengths in consecutive generations is approximately only a function of noise variables and c. Substituting $\alpha = 0.5$ in Eq. 3,

$$r(1) \approx \frac{\frac{1}{2}\frac{\sigma_{bd}^2 + 16\sigma_m^2}{3} + 4c\sigma_R^2}{\frac{\sigma_{bd}^2 + 16\sigma_m^2}{3} + 4\sigma_R^2}. \tag{4}$$

We find that $r(1)$ will be 0.5 in two cases—either $c = 0.5$ or $\sigma_R \ll \sqrt{\sigma_{bd}^2 + 16\sigma_m^2}$. Although either of these cases is possible in principle, strategies to regulate the radius across generations to be 0.5 are unknown. Thus, we conclude that the intrinsic variability of radius (σ_r) is much smaller than the noise in setting the division length. Most of the measured cell width variability has large contributions from measurement errors.

Until now, we have focused on models where cell volume is the relevant quantity the cell controls. The underlying assumption is that the rate of volume growth is proportional to the rate of protein synthesis and biomass accumulation. (17) observed that the cell surface area/biomass ratio remains constant during the cell cycle while density changes. This would mean that cell surface area, instead of the cell volume, is the appropriate proxy for biomass accumulation. However, our conclusions about the actual cell width variability being small remain unchanged even if we consider the surface area being controlled (supporting material section S4).

A striking observation in the experimental data is that the correlations between $L_{b,n}$ and $L_{d,n}$ closely agree with the conditional correlations $(L_{b,n}, L_{d,n}) \big| (R_{n-1}, R_n)$ (Tables 1, S1, and S2). Next, we use this observation and cell cycle model simulations to restrict the values of σ_R and c. In the simulations, we allow for a difference of 0.02 between the two correlation values. Most of the correlation differences obtained from experiments are within this limit (Tables 1, S1, and S2). We vary σ_R and c while keeping the measured values of radius CV (σ_{Rt}) and correlation between consecutive generations (c_t) fixed. We restrict the parameter space in the simulations such that the correlation between measurement noise in the radius in successive generations is between 0 and 1. For the division regulation strategy $f(V_b)$, we find that the correlation and conditional correlation are close for smaller values of σ_R (Fig. S7 *A*). We find that the measurement noise in width accounts for at least half of the total width variability. Similar results are obtained for cell cycle models beyond the $f(V_b)$ regulation strategy (Fig. S7, *B–D*).

Thus, the correlation and conditional correlation values bound the intrinsic cell width variability to be, at most, half the width variability observed in experiments.

CONCLUSION

Our previous work has shown that unaccounted sources of noise could lead to misinterpretation of the results of single-cell data analysis methods (18). (10) presented another example of the misinterpretation where width fluctuations would lead to a volume sizer being misinterpreted as a length adder (Figs. 1 *B* and 2, *A* and *B*). In a volume sizer, division happens when a certain sizer protein reaches a threshold amount irrespective of its initial amount at birth, and the protein biosynthesis is coupled to volume growth. Note that the underlying mechanism might not involve sensing the absolute protein numbers in the cell. The sizer proteins can be limited to a region of particular size. The rise in their concentration (or equivalently number) in this fixed region to a threshold amount can trigger division, as is found in fission yeast (19). In contrast, for a length adder, division happens when the protein accumulates a critical amount from birth and the protein biosynthesis is coupled to length growth. Thus, two differences arise between these two models: 1) what triggers division, is it the protein accumulation by a threshold amount or the protein number reaches a critical amount? 2) is the biomass accumulation related to length or volume?

Cell volume is also a suitable candidate to be the proxy for cell size. Biochemical reaction kinetics in the cell

depend on the concentration of reactant species, which is tied to the cell volume. (20) also showed that the average cell volume, rather than the cell length, surface area, and width, scaled as $e^{\langle\lambda\rangle(C+D)}$, where $\langle\lambda\rangle$ is the average growth rate and $C+D$ is the average time between the start of DNA replication and division. This observation can be explained by assuming that the average volume per origin of replication at the initiation of DNA replication is a constant, and division happens after a constant $C+D$ time from birth (21). In fast and intermediate growth conditions, the $C+D$ time was found to be 60 min (22). Since cell volume is the only cell geometry characteristic that follows the growth law, it is natural to assume that cell volume per origin is regulated at the start of DNA replication (23). Thus, assuming a volumetric control, we find from Eq. 4 that length and volume correlations can be the same if intrinsic radius variability is small compared to other fluctuations (division size or division ratio). Note that for a spherocylindrical cell geometry with negligible cell width variability, volume at birth or division is a linear function and not proportional to the cell length. However, cell cycle analysis frequently uses Pearson correlation coefficients, which are invariant under linear transformations. Hence, it is often equivalent to use volume or length in the cell cycle data analysis.

The mechanistic understanding of width control in bacteria is still an open question of research (24–27). Our results suggest that width control in *E. coli* is even more accurate than previously thought. We found that length correlations between birth and division and the conditional correlation between them when conditioned upon the radius in the mother cell and the current generation were extremely close in experimental data in multiple growth conditions from multiple studies (Tables 1, S1, and S2) (6,9,16). Using simulations of cell cycle models (Fig. S7), we found the range of intrinsic radius variability for which the above equality in correlations hold. The simulations indicate that the variance contribution of the measurement noise in the radius can be at least half of the total radius variability. If the CV of the radius is approximately 6% (Table S3), then our estimates put the CV of the intrinsic radius variability to be less than 4% (standard deviation $\approx$ 10 nm). In general, we conclude that the measured cell width variability is dominated by measurement noise. Hence, more accurate measurements, possibly using electron microscopy, are required to measure the actual (intrinsic) radius variability.

More broadly, adder behavior has been observed in multiple species across different domains of life (2,7,28–30). For a volume sizer to appear as a length adder requires fine-tuning cell cycle parameters, such as cell radius variability and noise in setting the division size. It is unlikely that the parameters are precisely controlled in these different organisms with different geometries and underlying cell cycle mechanisms. Why the adder behavior is so ubiquitous remains an open question.

ACKNOWLEDGMENTS

We thank Martin Howard, Sven van Teeffelen, and Sattar Taheri-Araghi for the useful feedback on the manuscript. A.A. and P.K. acknowledge support from NSF CAREER 1752024. A.A. is thankful for the generous support from the Clore Center for Biological Physics and ERC-CoG 2023 101125981.

AUTHOR CONTRIBUTIONS

A.A. and P.K. conceptualized the project, P.K. carried out the analysis, and P.K. and A.A. wrote the draft and reviewed and edited the manuscript. A.A. acquired funding.

DECLARATION OF INTERESTS

The authors declare no competing interests.

SUPPORTING MATERIAL

Supporting material can be found online at https://doi.org/10.1016/j.bpj.2025.03.019.

REFERENCES

1. Wang, P., L. Robert, …, S. Jun. 2010. Robust growth of *Escherichia coli*. *Curr. Biol.* 20:1099–1103.
2. Willis, L., and K. C. Huang. 2017. Sizing up the bacterial cell cycle. *Nat. Rev. Microbiol.* 15:606–620.
3. Jun, S., F. Si, …, M. Scott. 2018. Fundamental principles in bacterial physiology—history, recent progress, and the future with focus on cell size control: a review. *Rep. Prog. Phys.* 81:056601.
4. Ho, P.-Y., J. Lin, and A. Amir. 2018. Modeling cell size regulation: From single-cell-level statistics to molecular mechanisms and population-level effects. *Annu. Rev. Biophys.* 47:251–271.
5. Campos, M., I. V. Surovtsev, …, C. Jacobs-Wagner. 2014. A constant size extension drives bacterial cell size homeostasis. *Cell.* 159:1433–1446.
6. Taheri-Araghi, S., S. Bradde, …, S. Jun. 2015. Cell-size control and homeostasis in bacteria. *Curr. Biol.* 25:385–391.
7. Soifer, I., L. Robert, and A. Amir. 2016. Single-cell analysis of growth in budding yeast and bacteria reveals a common size regulation strategy. *Curr. Biol.* 26:356–361.
8. Witz, G., E. van Nimwegen, and T. Julou. 2019. Initiation of chromosome replication controls both division and replication cycles in *E. coli* through a double-adder mechanism. *Elife.* 8:e48063.
9. Si, F., G. Le Treut, …, S. Jun. 2019. Mechanistic origin of cell-size control and homeostasis in bacteria. *Curr. Biol.* 29:1760–1770.e7.
10. Facchetti, G., B. Knapp, …, M. Howard. 2019. Reassessment of the basis of cell size control based on analysis of cell-to-cell variability. *Biophys. J.* 117:1728–1738.
11. Amir, A. 2014. Cell size regulation in bacteria. *Phys. Rev. Lett.* 112:208102.
12. Baumgärtner, S., and I. M. Tolić-Nørrelykke. 2009. Growth pattern of single fission yeast cells is bilinear and depends on temperature and DNA synthesis. *Biophys. J.* 96:4336–4347.
13. Margolin, W. 2009. Sculpting the bacterial cell. *Curr. Biol.* 19:R812–R822.

14. Tanouchi, Y., A. Pai, …, L. You. 2015. A noisy linear map underlies oscillations in cell size and gene expression in bacteria. *Nature.* 523:357–360.

15. Kar, P., S. Tiruvadi-Krishnan, …, A. Amir. 2023. Using conditional independence tests to elucidate causal links in cell cycle regulation in *Escherichia coli*. *Proc. Natl. Acad. Sci. USA.* 120:e2214796120.

16. Colin, A., G. Micali, …, S. van Teeffelen. 2021. Two different cell-cycle processes determine the timing of cell division in *Escherichia coli*. *Elife.* 10:e67495.

17. Oldewurtel, E. R., Y. Kitahara, and S. van Teeffelen. 2021. Robust surface-to-mass coupling and turgor-dependent cell width determine bacterial dry-mass density. *Proc. Natl. Acad. Sci. USA.* 118:e2021416118.

18. Kar, P., S. Tiruvadi-Krishnan, …, A. Amir. 2021. Distinguishing different modes of growth using single-cell data. *Elife.* 10:e72565.

19. Facchetti, G., F. Chang, and M. Howard. 2017. Controlling cell size through sizer mechanisms. *Curr. Opin. Struct. Biol.* 5:86–92.

20. Zheng, H., P.-Y. Ho, …, C. Liu. 2016. Interrogating the *Escherichia coli* cell cycle by cell dimension perturbations. *Proc. Natl. Acad. Sci. USA.* 113:15000–15005.

21. Donachie, W. D. 1968. Relationship between cell size and time of initiation of DNA replication. *Nature.* 219:1077–1079.

22. Cooper, S., and C. E. Helmstetter. 1968. Chromosome replication and the division cycle of *Escherichia coli* Br. *J. Mol. Biol.* 31:519–540.

23. Wallden, M., D. Fange, …, J. Elf. 2016. The synchronization of replication and division cycles in individual *E. coli* cells. *Cell.* 166:729–739.

24. Furchtgott, L., N. S. Wingreen, and K. C. Huang. 2011. Mechanisms for maintaining cell shape in rod-shaped Gram-negative bacteria. *Mol. Microbiol.* 81:340–353.

25. Amir, A., and S. van Teeffelen. 2014. Getting into shape: How do rod-like bacteria control their geometry? *Syst. Synth. Biol.* 8:227–235.

26. Nguyen, L. T., J. C. Gumbart, …, G. J. Jensen. 2015. Coarse-grained simulations of bacterial cell wall growth reveal that local coordination alone can be sufficient to maintain rod shape. *Proc. Natl. Acad. Sci. USA.* 112:E3689–E3698.

27. Wong, F., L. D. Renner, …, A. Amir. 2017. Mechanical strain sensing implicated in cell shape recovery in *Escherichia coli*. *Nature Microbiology.* 2:1–8.

28. Logsdon, M. M., P.-Y. Ho, …, B. B. Aldridge. 2017. A parallel adder coordinates mycobacterial cell-cycle progression and cell-size homeostasis in the context of asymmetric growth and organization. *Curr. Biol.* 27:3367–3374.e7.

29. Eun, Y.-J., P.-Y. Ho, …, A. Amir. 2018. Archaeal cells share common size control with bacteria despite noisier growth and division. *Nat. Microbiol.* 3:148–154.

30. Cadart, C., S. Monnier, M. Piel…, 2018. Size control in mammalian cells involves modulation of both growth rate and cell cycle duration. *Nat. Commun.* 9:3275.

Biophysical Journal, Volume *124*

Supplemental information

Are cell length and volume interchangeable in cell cycle analysis?

Prathitha Kar and Ariel Amir

Supplemental Figures

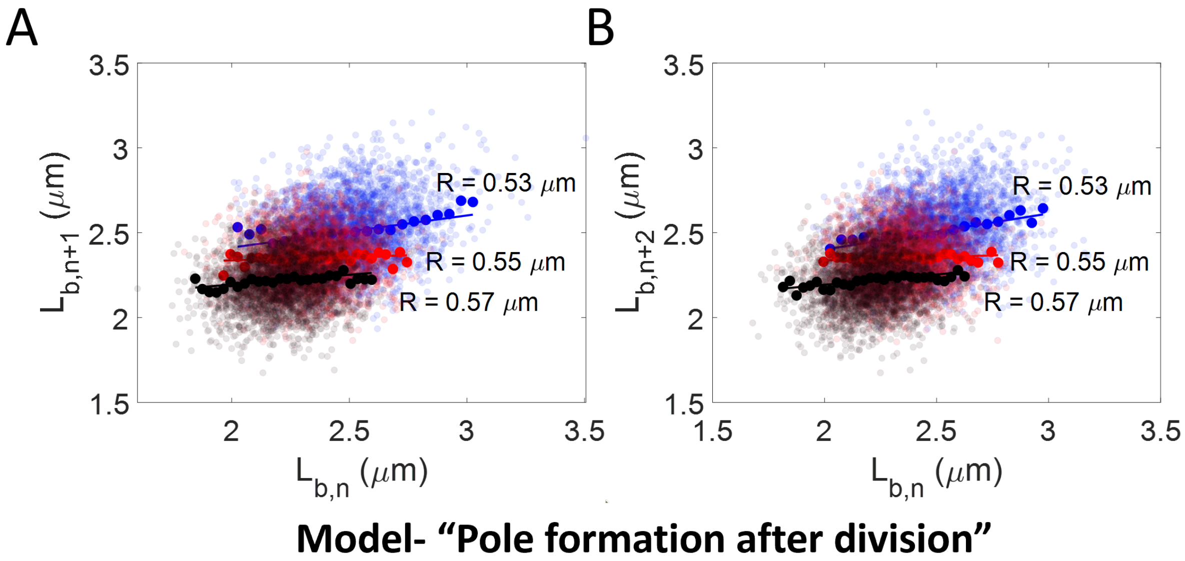

Figure S1: **Model "Pole formation after division" - A-B.** We simulate the model described in Figure 2 of the main text. We arrange the simulated dataset in ascending order of radius R and divide it into three groups. For each group (with a different average radius), we plot the **A.** $L_{b,n+1}$ vs $L_{b,n}$ plot. **B.** $L_{b,n+2}$ vs $L_{b,n}$ plot. These plots illustrate Simpson's paradox.

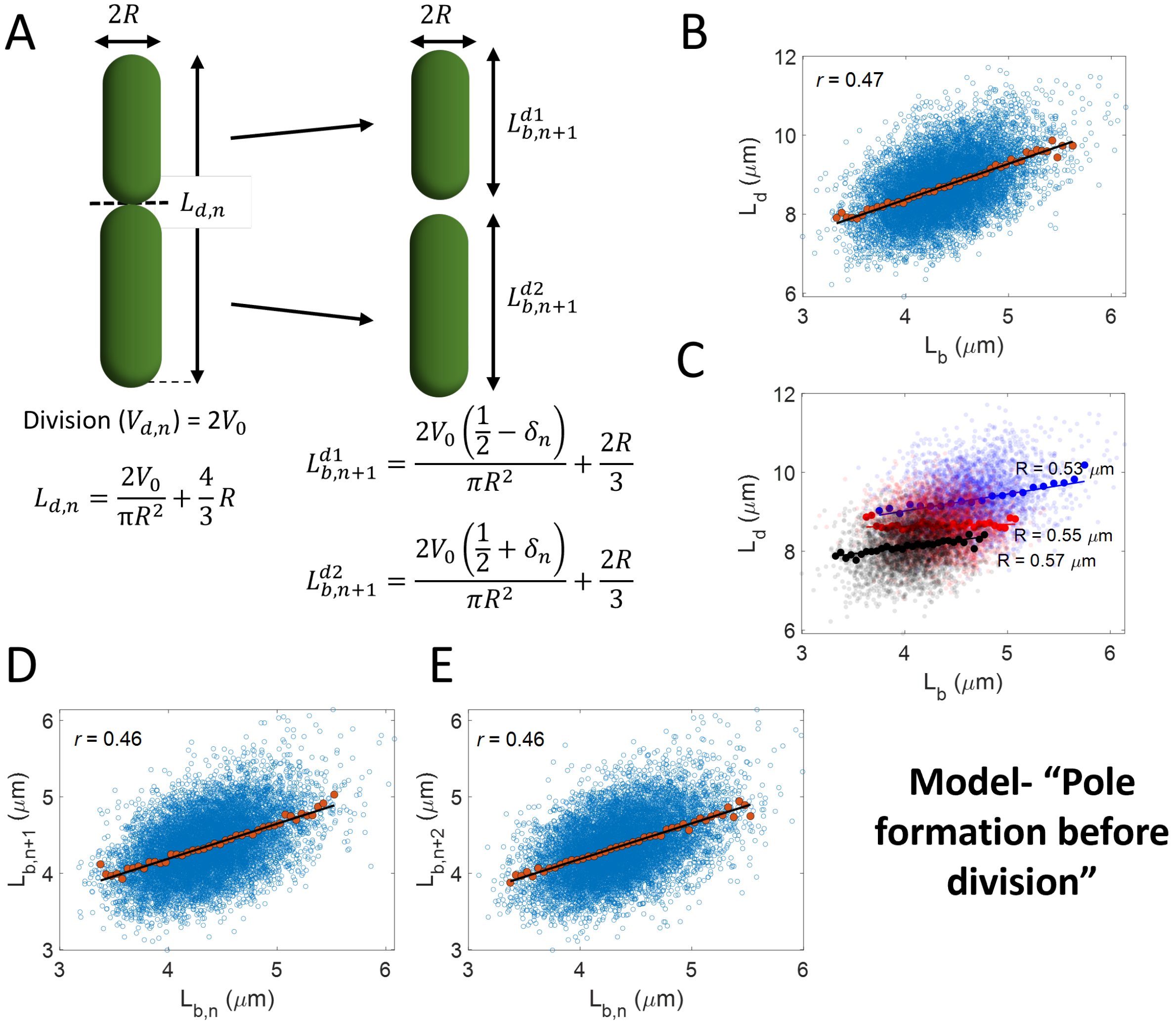

Figure S2: **Model "Pole formation before division" - A.** Schematic of the model proposed in Section "Correlation structure in a cell division model where the new pole forms at mid-cell before division" of the main text. The model borrows certain aspects from the model in Figure 2A of the main text. In both models, cells have a spherocylindrical geometry and they divide upon reaching a critical volume. The radius is fixed for a particular lineage and varies between lineages. However, unlike the model in Figure 2A, there are already two fully formed cells just before cell division. The cells divide symmetrically, on average, by volume. **B-E.** Simulations of the model in Figure S2A are carried out for 10000 cell lineages and over 25 generations. For the simulations, **B.** we plot L_d vs L_b plot. The correlation r (top left) points to a near-adder model. **C.** We plot L_d vs L_b for small subsets of R. We arrange the simulated dataset in ascending order and divide it into three groups. For each group (with a different average radius), we plot the L_d vs L_b plot. The plot shows Simpson's paradox mentioned in Figure 1B. **D.** Length at birth in generation $n+1$ vs generation n is plotted. **E.** Length at birth in generation $n+2$ vs generation n is plotted. The correlation values are identical and consistent with Eq. S28. In all the plots, the cloud is the raw data, the dots represent the binned data, and the line is the best linear fit.

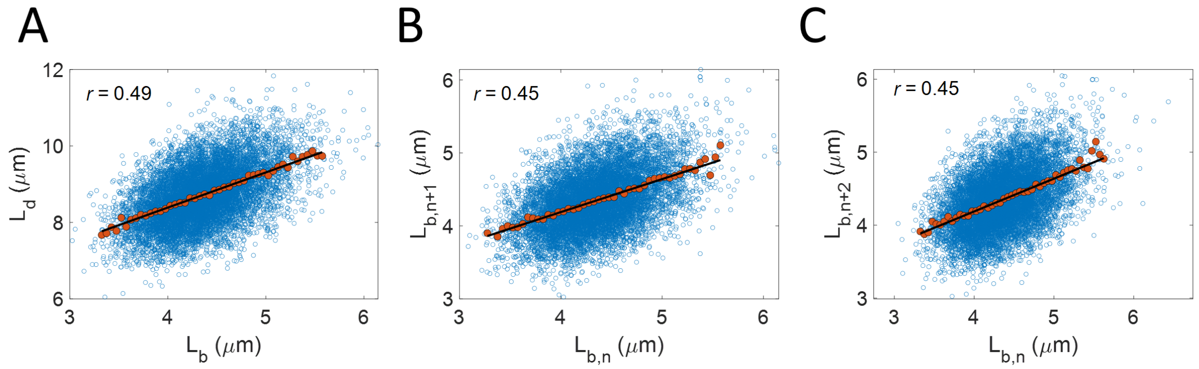

Figure S3: **A-C.** The simulation results presented here correspond to the cell cycle model in Figure S2A. The only difference between the two models is that cells divide symmetrically, on average, by length instead of volume. The length at birth for the daughter cell $L_{b,n+1} = L_{d,n}(\frac{1}{2} + \delta_n)$. Simulations of the model are carried out for 10000 cell lineages and over 25 generations. For the simulations, **A.** we plot L_d vs L_b plot. The correlation r (top left) points to a near-adder model. **B.** Length at birth in generation $n + 1$ vs generation n is plotted. **C**. Length at birth in generation $n + 2$ vs generation n is plotted. The correlation values in all the plots are close to that in Figure S2. In all the plots, the cloud is the raw data, the dots represent the binned data, and the line is the best linear fit.

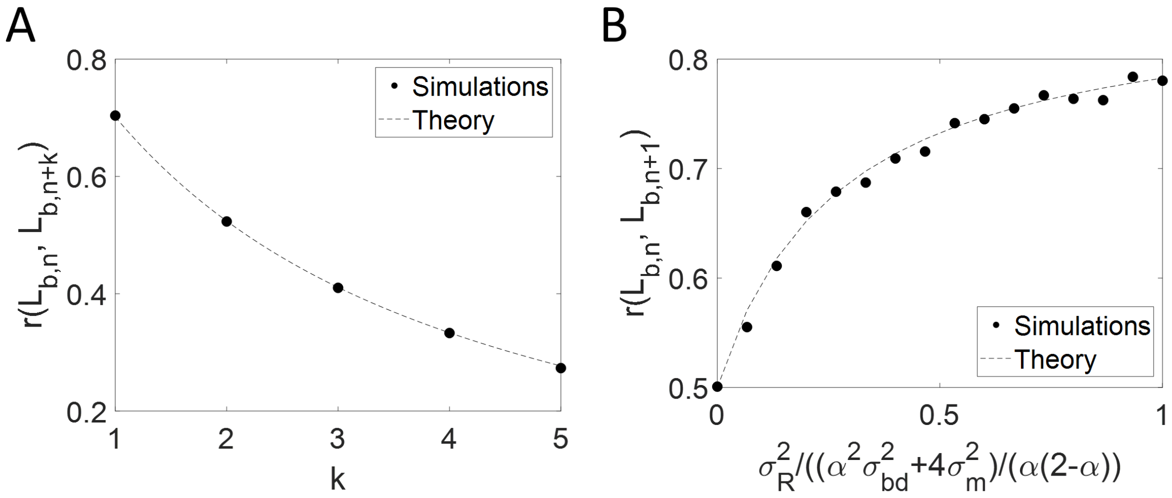

Figure S4: **Model "Changing radii in a lineage" - A-B.** We simulate the model described in Figure 3A of the main text. **A.** We find that the Pearson correlation coefficients between the lengths at birth in the nth generation ($L_{b,n}$) and $n+k^{th}$ generation ($L_{b,n+k}$) obtained from simulations (black dots) match the theoretical predictions (black dashed line) shown in Eq. 3 of the main text/Eq. S27 of the SI text. **B.** The coefficient of variation (CV) of the cell radius (σ_R) is varied in the simulations keeping the value of $\alpha = \frac{1}{2}$ (volume adder), size additive division size noise ($\sigma_{bd} = 0.19$, and noise in the division ratio ($\sigma_m = 0.03$) fixed. We find that the correlations between birth lengths in consecutive generations obtained from simulations (black dots) are consistent with the theoretical predictions (black dashed line) of Eq. 3 of the main text/Eq. S27 of the SI text for different values of σ_R.

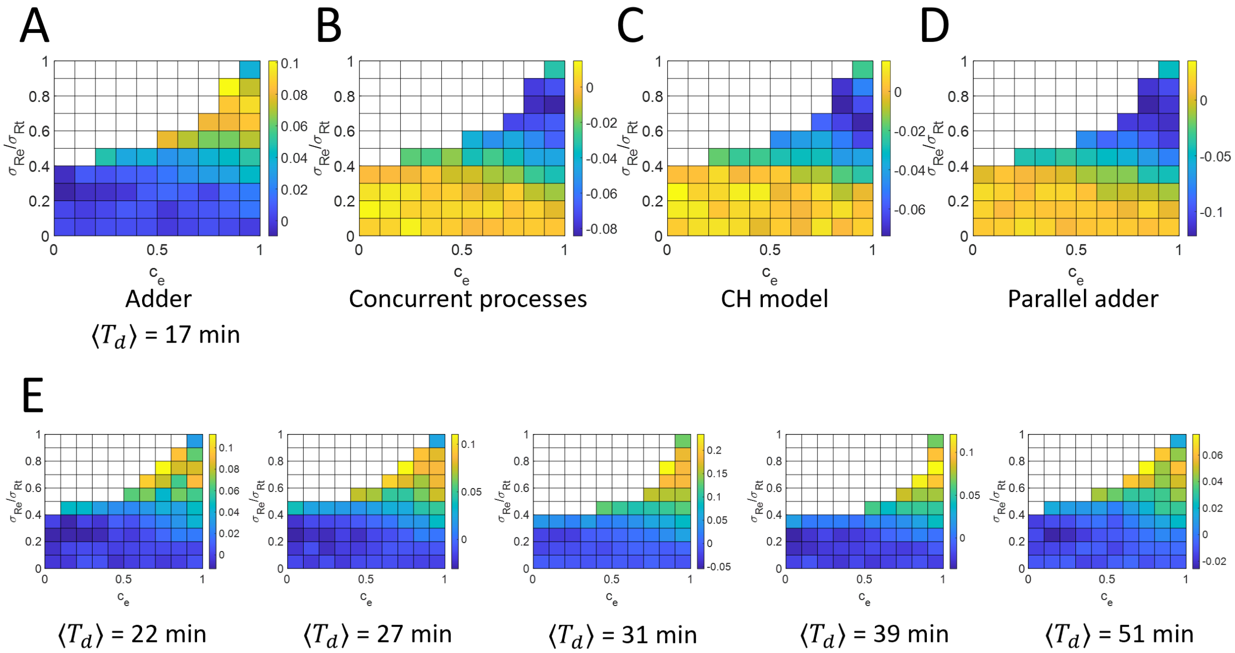

Figure S5: **Verifying the conditional correlation method to infer volume regulation strategies. A-D.** Differences between the correlation obtained from V_d vs V_b plot and $L_{d,n}|(R_{n-1}, R_n)$ vs $L_{b,n}|(R_{n-1}, R_n)$ plot are shown. In the section "Determining the volume cell cycle regulation strategy" of the main text, we put forth that the conditional correlation between the length at birth ($L_{b,n}$) and length at division ($L_{d,n}$) when conditioned upon radius in the current generation (R_n) and the previous generation R_{n-1} is an appropriate method to study cell cycle regulation mechanisms despite width fluctuations. We simulate various cell cycle models with each plot representing a different model regulating the division size. In each of these simulations, we vary the measurement error in the cell radius measurements (σ_{Re}) and the correlation between the radius measurement errors in consecutive generations such that the correlation between the actual radius in consecutive generations is between 0 and 1. The white areas in the plots are the regions that do not follow this condition. The measured coefficient of variation (CV) of the radius (σ_{Rt}) and the measured correlations between the radius in consecutive generations (c_t) are fixed in the simulations. The differences in the actual and inferred volume regulation strategies are plotted. **A.** The division strategy is the adder model ($\alpha = \frac{1}{2}$). The values in the plot correspond to $\frac{1}{2} - \alpha_{infer}$ where α_{infer} is obtained using the slope of $L_{d,n}|(R_{n-1}, R_n)$ vs $L_{b,n}|(R_{n-1}, R_n)$ plot (Section S1.2.2). **B-D.** The plot values correspond to the difference between the Pearson correlation coefficients of V_d vs V_b plot and $L_{d,n}|(R_{n-1}, R_n)$ vs $L_{b,n}|(R_{n-1}, R_n)$ plot. **B.** The division strategy is the concurrent processes model [S1] where both birth and the start of DNA replication control the division volume (see S1.2.2 for details) **C-D.** The division volume is solely controlled by the volume at the start of DNA replication. **C.** Division happens after a constant time from the initiation of DNA replication [S2]. **D.** Division happens after an addition of constant volume per origin of replication from the initiation of DNA replication. **E.** The simulations are carried out using parameters derived from different growth conditions in [S3]. The simulation model is the same as in Figure S5A.

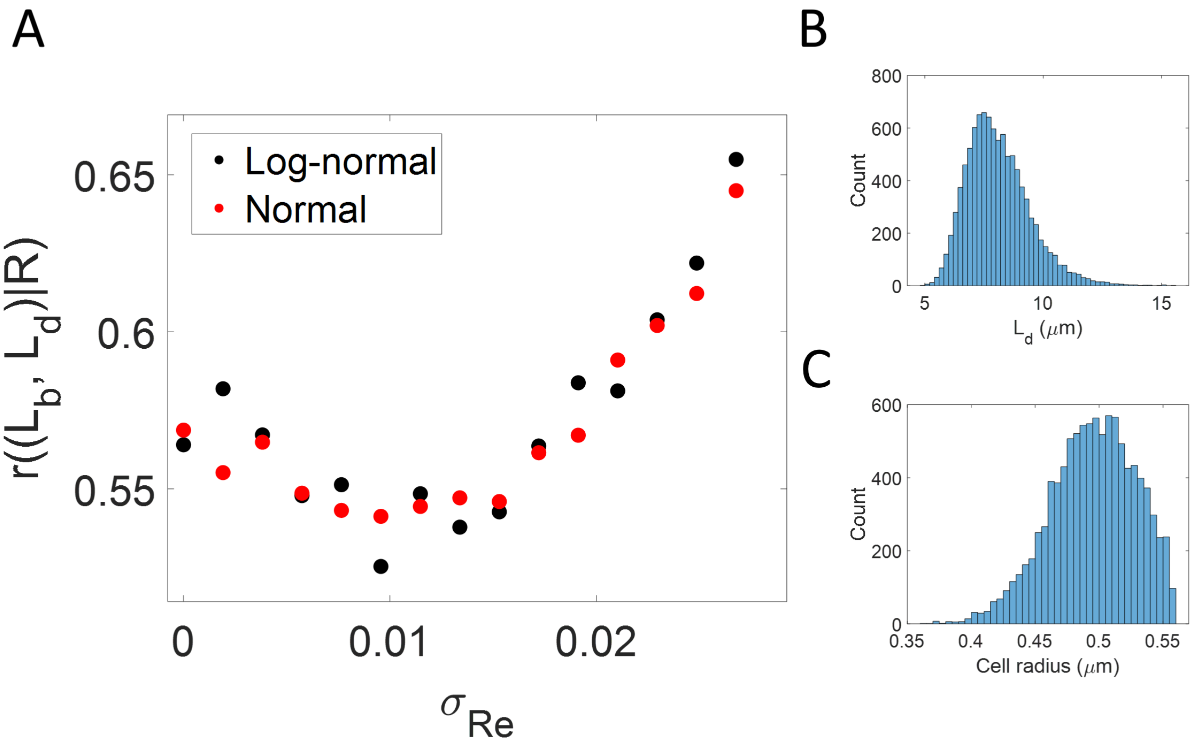

Figure S6: **A.** Conditional correlation $r(L_b, L_d|R)$ as a function of the standard deviation of the error in radius measurements. **B-C.** The simulations shown in (A) assumed that the cell lengths are distributed log-normally and radius measurements are restricted at higher values. We show the distribution of **B.** cell length at division, **C.** cell radius, from one such simulation.

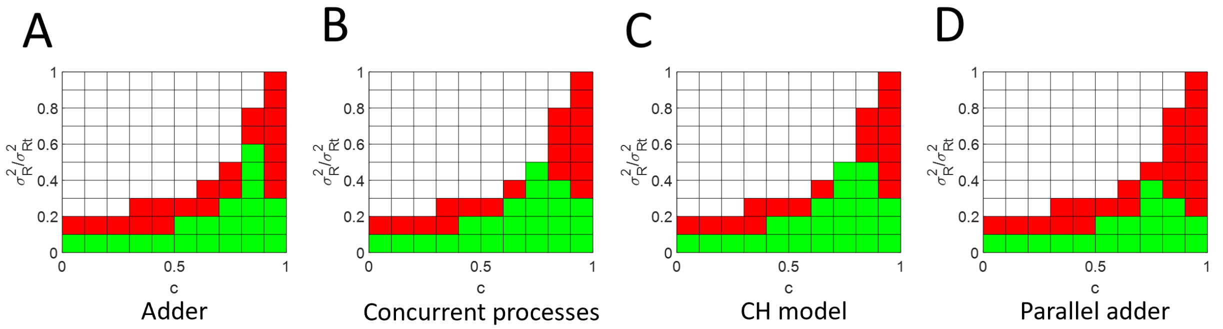

Figure S7: **A-D.** The differences between the conditional correlation $(L_{b,n}, L_{d,n}|(R_{n-1}, R_n)$ and correlation $(L_{b,n}, L_{d,n})$ are calculated. If the absolute magnitude of the difference is less than 0.02, the value is set to 1 (green) or else zero (red). The plots are made for simulations of the model in Figure 3A of the main text. The difference between the four plots is the cell cycle model regulating the division size. The measured coefficient of variation (CV) of the radius (σ_{Rt}) and the measured correlations between the radius in consecutive generations (c_t) are fixed in the simulations. The standard deviation of the actual radius fluctuations (σ_R), and the correlation between the actual radii in consecutive generations (c) is varied such that the correlation between the measurement noise in radius in consecutive generations (c_e) is between 0 and 1. The white areas in the plots are the regions that do not follow this condition. **A.** Division volume is determined by the birth volume. **B.** The division strategy is the concurrent processes model [S1] where both birth and the start of DNA replication control the division volume (see S1.2.2 for details) **C-D.** The division volume is solely controlled by the volume at the start of DNA replication. **C.** Division happens after a constant time from the initiation of DNA replication [S2]. **D.** Division happens after the addition of constant volume per origin of replication from the initiation of DNA replication.

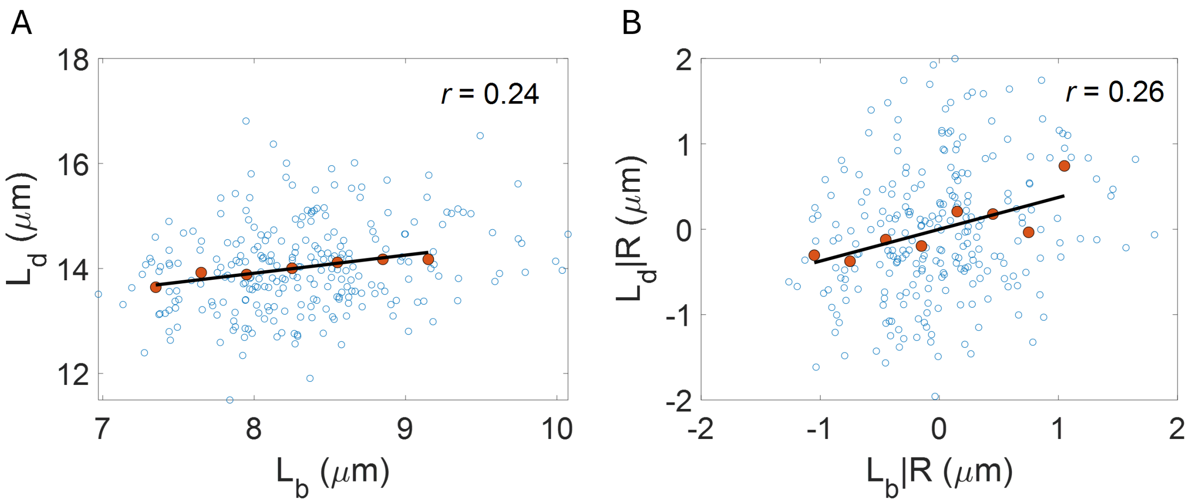

Figure S8: **A-B.** We analyze WT fission yeast data from Facchetti *et al.* [S4] (number of cells, N = 256). For the dataset, we make **A.** L_d vs L_b plot. **B.** Conditional correlation between L_b and L_d conditioned on R, $L_d|R$ vs $L_b|R$ plot. r is the Pearson correlation coefficient in both these plots.

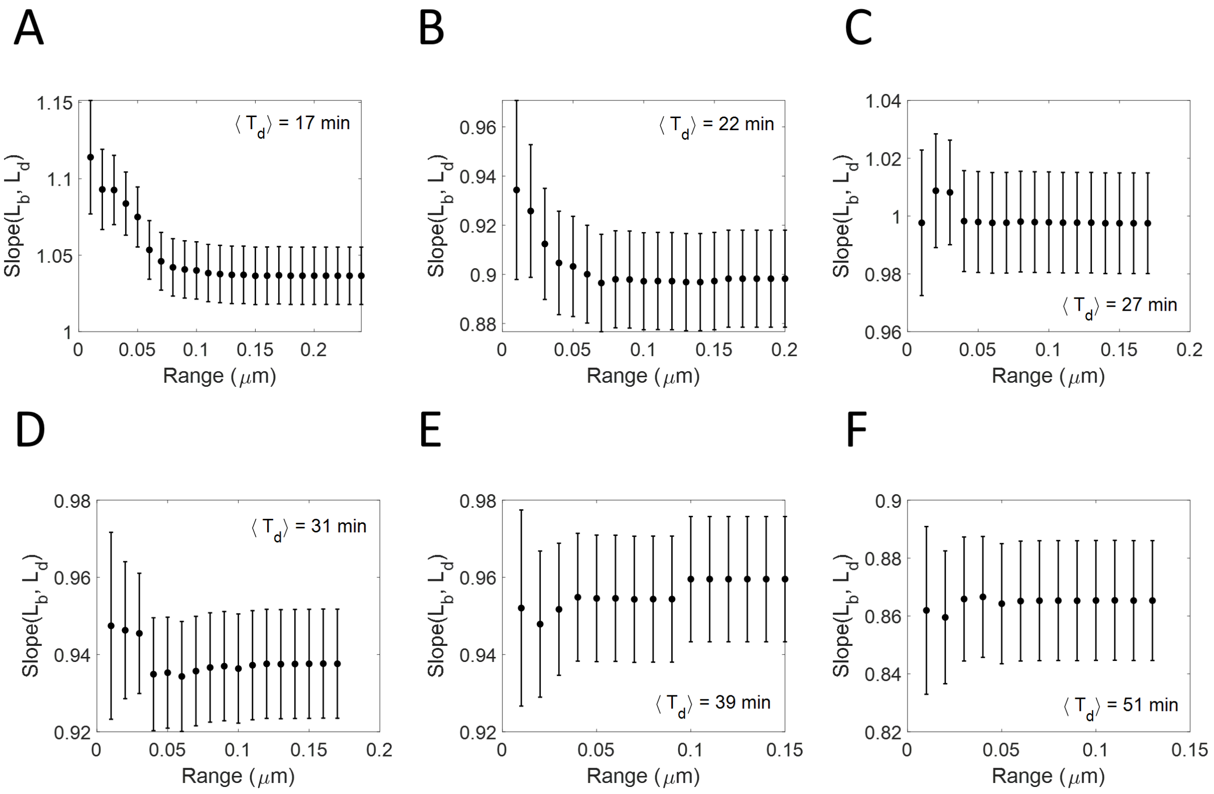

Figure S9: **A-F.** We analyzed *E. coli* data from six different growth media [S3]. For each growth media, we restricted the dataset to those points where the cell radii fell within a particular range around the mean radius. This mimics the decreased radius variability associated with conditional correlation when conditioned upon cell radius. For different range values, we plot the slope of the best linear fit of L_d vs L_b plot. We find that the slope is close to one in all these plots, thus, pointing to an adder model.

Supplemental Tables

$\langle \mathbf{T_d} \rangle$ (min)	No. of cells	$(L_{b,n}, L_{d,n})$	$(L_{b,n}, L_{d,n})\|(R_{n-1}.R_n)$	$(L_{b,n}, L_{b,n+1})$
74	298	0.50 (0.41, 0.58)	0.48 (0.41, 0.58)	0.34 (0.23 0.43)
82	796	0.62 (0.57, 0.66)	0.62 (0.58, 0.66)	0.56 (0.51 0.61)
110	430	0.56 (0.49, 0.62)	0.54 (0.47, 0.61)	0.49 (0.42 0.56)

Table S1: Pearson correlation coefficients along with their 95% confidence intervals (CI) are shown for three datasets of untreated *E. coli* cells in Ref. [S5] with mean generation times, $\langle T_d \rangle$. Correlation between lengths at birth ($L_{b,n}$) and division ($L_{d,n}$), lengths at birth in consecutive generations ($L_{b,n}$ and $L_{b,n+1}$), and conditional correlation, $(L_{b,n}, L_{d,n})|(R_{n-1}, R_n)$, are shown. R_n is the cell radius when the cell divides in generation n.

$\langle \mathbf{T_d} \rangle$ (min)	No. of cells	$(L_{b,n}, L_{d,n})$	$(L_{b,n}, L_{d,n})\|(R_{n-1}.R_n)$	$(L_{b,n}, L_{b,n+1})$
29	757	0.55 (0.49, 0.59)	0.54 (0.49, 0.59)	0.54 (0.49 0.59)
43	854	0.52 (0.47, 0.56)	0.53 (0.47, 0.57)	0.56 (0.52 0.61)
52	1482	0.50 (0.47, 0.54)	0.53 (0.49, 0.56)	0.49 (0.45 0.53)
64	992	0.51 (0.46, 0.55)	0.54 (0.49, 0.58)	0.44 (0.39 0.49)
113	925	0.60 (0.56, 0.64)	0.59 (0.55, 0.63)	0.63 (0.59 0.67)
194	680	0.49 (0.43, 0.54)	0.48 (0.42, 0.54)	0.44 (0.37 0.50)

Table S2: Pearson correlation coefficients along with their 95% confidence intervals (CI) are shown for *E. coli* growing in six different steady-state growth conditions [S6] with mean generation times, $\langle T_d \rangle$. Correlation between lengths at birth (L_b) and division (L_d), lengths at birth in consecutive generations ($L_{b,n}$ and $L_{b,n+1}$), and conditional correlation, $(L_{b,n}, L_{d,n})|(R_{n-1}, R_n)$, are shown.

**Media**	$\langle \mathbf{T_d} \rangle$ **(min)**	$\mathbf{V_0}$ $\mu\mathbf{m^3}$	$\sigma_{\mathbf{bd}}$	$\sigma_{\mathbf{m}}$	$\mathbf{R_0}$ $\mu\mathbf{m}$	$\sigma_{\mathbf{R}}$	**c**	α	**r(1) - Eq. 3, main text**
Tryptic soy broth	17	2.77	0.19	0.03	0.49	0.08	0.86	0.46	0.72
Synthetic rich	22	1.70	0.17	0.03	0.42	0.07	0.85	0.55	0.67
glucose + 12 a.a.	27	1.04	0.09	0.02	0.35	0.05	0.84	0.50	0.73
glucose + 6 a.a.	31	0.77	0.04	0.02	0.34	0.07	0.90	0.52	0.82
glucose	39	0.58	0.10	0.02	0.32	0.06	0.91	0.52	0.76
sorbitol	51	0.46	0.14	0.04	0.27	0.06	0.84	0.57	0.62

Table S3: Model parameter values substituted to Eq. 3 of the main text and $r(1)$ values obtained from it. The model parameters are obtained using experimental data from Ref. [S3] and explained in SI text.

S1 Rejecting models where volume sizer leads to length adder

S1.1 Volume sizer model in Ref. [S4]

Previous works [S4, S7] have shown that unaccounted noise variables can lead to misinterpretations of regression and correlation values. The main text focuses on one such example [S4] where the cell width fluctuations cause the actual underlying mechanism of a volume sizer (cell divides upon reaching a critical volume) to appear as a length adder (division happens upon constant length increment from birth). We will demonstrate it in this section by analytically calculating the expressions for the correlations between various cell cycle variables (such as length at birth and length at division) using simple cell cycle regulation models. Note that some of the calculations are shown here to introduce the model and motivate the question but these results are also presented in Refs. [S8], [S4], and [S7]. Further, we will show model correlation predictions that do not agree with the experimental data.

We use a simplistic model of cell division that was proposed in Ref. [S9]. In the model, cells divide when they reach a volume V_d determined solely by their birth volume, V_b. Mathematically, we model this size regulation strategy as $V_d = f(V_b)$, where $f(V_b) = 2(1-\alpha)V_b + 2\alpha V_0$. α denotes the strength of the size regulation strategy and V_0 is the average cell volume at birth. $\alpha = \frac{1}{2}$ corresponds to the above-mentioned adder strategy while $\alpha = 1$ points to the sizer strategy. To account for the stochasticity in the cell division process, we introduce a size additive noise in addition to $f(V_b)$,

$$V_d = 2(1-\alpha)V_b + 2\alpha V_0(1 + \zeta_s(0, \sigma_{bd})). \tag{S1}$$

In the simulations, the noise $\zeta_s(0, \sigma_{bd})$ is drawn from a normal distribution with zero mean and standard deviation of σ_{bd}. ζ_s in successive generations are independent of each other.

Note that we use a size-additive noise due to ease of calculations, however, the nature of division noise (time or size additive) does not affect the results qualitatively [S7].

Assuming a spherocylinder geometry for the rod-shaped *E. coli* cells, we find the length at division (L_d) to be,

$$L_d = \frac{V_d}{\pi R^2} + \frac{2}{3}R, \tag{S2}$$

where R is the cell radius. The cell radius is assumed to fluctuate on timescales greater than the doubling time such that cells within a few generations of a particular lineage will have the same radius while those from different lineages (with common ancestors multiple generations ago) will have different radii. The radius for different lineages is drawn from a normal distribution with R_0 mean and coefficient of variation (CV) $= \sigma_R$ in the simulations.

The cell divides into two symmetrical cells on average (mean $= 1/2$). The inaccuracy in setting the division plane at the end of nth generation of a lineage is δ_n which has a mean $= 0$ and standard deviation $= \sigma_m$. The length at birth in the $n+1^{th}$ generation ($L_{b,n+1}$) is related to the length at division in the previous (n^{th}) generation ($L_{d,n}$) as,

$$L_{b,n+1} = \frac{L_{d,n}}{2}(1+\delta_n) + \frac{R}{3}. \tag{S3}$$

The $\frac{R}{3}$ term in Eq. S3 is added to denote the addition of a hemispherical pole upon the contraction of the division plane while conserving the cell volume.

Throughout our calculations, we will assume that fluctuations in cell width ($\zeta_R(0, \sigma_R)$) and the division noise ($\zeta_s(0, \sigma_{bd})$) are small. On substituting $\alpha = 1$ in Eq. S1- corresponding to volume sizer- and rearranging Eq. S2 by keeping noise terms up to first order, we find the cell length at division to be,

$$L_{d,n} \approx \frac{2V_0}{\pi R_0^2} + \frac{2}{3}R_0 + \frac{2V_0}{\pi R_0^2}\zeta_{s,n} + \zeta_R\left(\frac{2}{3}R_0 - \frac{4V_0}{\pi R_0^2}\right). \tag{S4}$$

Note that $\zeta_{s,n}$ is the value of ζ_s in the nth generation. The variance of length at division for a population of cells from different lineages is,

$$\sigma_d^2 = \frac{9V^2\sigma_{bd}^2 + 4(\pi R_0^3 - 3V)^2\sigma_R^2}{(3\pi R_0^2)^2}, \tag{S5}$$

where $V = 2V_0$. We assumed that the division noise in a cell cycle is independent of the width fluctuations. Eq. S5 is identical to that in [S4] except for the additional σ_e^2 corresponding to the variance contribution from measurement errors of cell lengths.

To calculate the birth length, we simplify Eq. S3 keeping the first-order noise terms,

$$L_{b,n+1} \approx \frac{V}{2\pi R_0^2} + \frac{2}{3}R_0 + \frac{V}{2\pi R_0^2}\zeta_{s,n} + \zeta_R\left(\frac{2}{3}R_0 - \frac{V}{\pi R_0^2}\right) + \delta_n\left(\frac{V}{2\pi R_0^2} + \frac{R_0}{3}\right). \tag{S6}$$

Assuming that δ_n is independent of both $\zeta_{s,n}$ and ζ_R, the variance of the birth length for a cell population is,

$$\sigma_b^2 = \left(\frac{V}{2\pi R_0^2}\right)^2\sigma_{bd}^2 + \left(\frac{2}{3}R_0 - \frac{V}{\pi R_0^2}\right)^2\sigma_R^2 + \left(\frac{V}{2\pi R_0^2} + \frac{R_0}{3}\right)^2\sigma_m^2. \tag{S7}$$

S1.1.1 L_d vs L_b plot is consistent with adder model predictions

Next, we will show that such an apparent adder correlation in length can arise due to cell width fluctuations in a volume sizer model (Figure 1 in the main text and Ref. [S4]). The slope of the best linear fit for L_d vs L_b plot is one for the adder model.

The slope of L_d vs L_b plot can be calculated as,

$$m_{bd} = \frac{Cov(L_{b,n}, L_{d,n})}{\sigma_b^2}, \tag{S8}$$

where $Cov(L_{b,n}, L_{d,n})$ is the covariance between L_b and L_d.

Using the expressions of $L_{b,n}$ and $L_{d,n}$ from Eqs. S6 and S4, respectively,

$$Cov(L_{b,n}, L_{d,n}) = \left(\frac{2}{3}R_0 - \frac{V}{\pi R_0^2}\right)\left(\frac{2}{3}R_0 - \frac{2V}{\pi R_0^2}\right)\sigma_R^2. \quad \text{(S9)}$$

On substituting Eq. S9 into Eq. S8, we obtain,

$$m_{bd} = \frac{\left(\frac{2}{3}R_0 - \frac{V}{\pi R_0^2}\right)\left(\frac{2}{3}R_0 - \frac{2V}{\pi R_0^2}\right)\sigma_R^2}{\left(\frac{V}{2\pi R_0^2}\right)^2 \sigma_{bd}^2 + \left(\frac{2}{3}R_0 - \frac{V}{\pi R_0^2}\right)^2 \sigma_R^2 + \left(\frac{V}{2\pi R_0^2} + \frac{R_0}{3}\right)^2 \sigma_m^2}. \quad \text{(S10)}$$

If the variance contribution from measurement errors is included in σ_b^2, the slope in Eq. S10 will be identical to that in Ref. [S4]. Using values from Ref. [S4] - $V = 3.77\ \mu m^3$; $R_0 = 0.55\ \mu m$; $\sigma_{bd} = 0.065$; $\sigma_R = 0.035$; $\sigma_m = 0.032$- we obtain the slope to be 0.89. This is very close to the prediction for length adder (slope = 1). Note that measurement errors in length only change the variance of L_b i.e., the denominator in Eq. S10. The slope changes only slightly (= 0.86) upon including the measurement error to birth lengths (standard deviation = σ_e = 0.04 μm).

S1.1.2 Correlation between birth lengths in successive generations do not agree with experiments

Next, we calculate the correlation between the birth length of the nth cell cycle ($L_{b,n}$) and the birth length k generations later ($L_{b,n+k}$). An ideal cell cycle model should be able to explain the correlations between all cell cycle variables including between $L_{b,n}$ and $L_{b,n+k}$. In this section, we will show that while the L_d vs L_b plot points to a length adder, the correlation between $L_{b,n}$ and $L_{b,n+k}$ does not agree with it when the underlying control is volume sizer.

Similar to Eq. S6, the length at birth k generations after is,

$$L_{b,n+k} \approx \frac{V}{2\pi R_0^2} + \frac{2}{3}R_0 + \frac{V}{2\pi R_0^2}\zeta_{s,n+k-1} + \zeta_R\left(\frac{2}{3}R_0 - \frac{V}{\pi R_0^2}\right) + \delta_{n+k-1}\left(\frac{V}{2\pi R_0^2} + \frac{R_0}{3}\right). \quad \text{(S11)}$$

We will quantify correlations between X and Y using Pearson correlation coefficients,

$$r_{xy} = \frac{Cov(x, y)}{\sigma_x \sigma_y}, \tag{S12}$$

where σ_x and σ_y are the standard deviations of X and Y, respectively. At steady state, the standard deviation of $L_{b,n}$ and $L_{b,n+k}$ are equal to σ_b.

Using Eqs. S6 and S11, we obtain,

$$Cov(L_{b,n}, L_{b,n+k}) = \left(\frac{2}{3}R_0 - \frac{V}{\pi R_0^2}\right)^2 \sigma_R^2. \tag{S13}$$

The Pearson correlation coefficient between $L_{b,n}$ and $L_{b,n+k}$ is,

$$r = \frac{\left(\frac{2}{3}R_0 - \frac{V}{\pi R_0^2}\right)^2 \sigma_R^2}{\left(\frac{V}{2\pi R_0^2}\right)^2 \sigma_{bd}^2 + \left(\frac{2}{3}R_0 - \frac{V}{\pi R_0^2}\right)^2 \sigma_R^2 + \left(\frac{V}{2\pi R_0^2} + \frac{R_0}{3}\right)^2 \sigma_m^2}. \tag{S14}$$

The equation is the same for any value of k for which the radius can be assumed to be constant. Using the values from Ref. [S4], $r = 0.43$ (for $\sigma_e = 0$) and $r = 0.41$ (for $\sigma_e = 0.04$ μm). We also verify the results using simulations in Figure 2F of the main text.

S1.2 Model 2: Length growth at mid-plane before cell division in *Escherichia coli*

The model in Fachetti *et al.* was inspired by cell growth studies in fission yeast [S4]. However, unlike fission yeast, *E. coli* cells do not undergo an abrupt increase in length before birth. *E. coli* cells start constricting at the mid-cell and form the new hemispherical poles before the division event [S10]. In this section, we will explore a cell cycle model where there are two fully formed daughter cells at division. Further, we will relax the assumption that the cell width is the same in a lineage. We will allow the radius to vary during the cell cycle

but the radius across generations might still be correlated. The schematic of the cell cycle model motivated here is shown in Figure 3A of the main text.

The division length and birth length are calculated differently as compared to the previous section. According to this model, the cell forms two daughter cells with two hemispherical poles. Thus, the length at division is,

$$L_{d,n} = \frac{V_{d,n}}{\pi R_n^2} + \frac{4}{3}R_n, \tag{S15}$$

where R_n is the cell radius at the time of division of generation n.

The positioning of the septum is partly determined by the Min system oscillations which divide the cells, on average, symmetrically in length [S11, S12]. For this discussion, we consider cells dividing into two symmetrical cells, on average, by volume. However, the choice of division by length or volume is irrelevant. We will obtain the same qualitative results if cells were dividing by length as shown in Figure S3. Thus, the volume at birth in the next generation $n+1$ is,

$$V_{b,n+1} = V_{d,n}\left(\frac{1}{2} + \delta_n\right), \tag{S16}$$

where δ_n is the noise in volume division ratio $= \frac{V_{b,n+1}}{V_{d,n}}$ for a cell dividing in generation n. It has a mean $= 0$ and standard deviation $= \sigma_m$. The length at birth in generation $n+1$ ($L_{b,n+1}$) is,

$$L_{b,n+1} = \frac{V_{b,n+1}}{\pi R_n^2} + \frac{2}{3}R_n. \tag{S17}$$

The underlying assumption in Eq. S16 is that the radius doesn't change during the division event but can undergo variation during a cell cycle such that the radius at the end of $n+1^{th}$ generation is,

$$R_{n+1} = R_0 + c(R_n - R_0) + \sqrt{1-c^2}R_0\zeta_{R,n+1}. \tag{S18}$$

Here, R_0 is the mean radius, c is the correlation between radii in consecutive generations,

and $\zeta_{R,n+1}$ is the noise in cell radius. $\zeta_{R,n}$ is assumed to be normally distributed with mean 0, variance $(\langle\zeta_{R,n}^2\rangle) = \sigma_R^2$ and the noise is independent of that in a different generation i.e., $\langle\zeta_{R,n}\zeta_{R,n+1}\rangle = 0$.

On Taylor expanding and keeping the first-order terms, $L_{b,n}$ in Eq. S17 is found to be,

$$L_{b,n} \approx \frac{V_{b,n}}{\pi R_0^2} + \frac{2}{3}R_0 + \left(\frac{2}{3}R_0 - \frac{2V_0}{\pi R_0^2}\right)\zeta'_{R,n-1}, \tag{S19}$$

where $\zeta'_{R,n} = c\frac{R_{n-1}-R_0}{R_0} + \sqrt{1-c^2}\zeta_{R,n}$. Using Eq. S18, we find the covariance, $Cov(\zeta'_{R,n-1}, \zeta'_{R,n}) = c\sigma_R^2$.

Similarly, keeping till the first order terms, $L_{d,n}$ is calculated by substituting Eq. S1 into Eq. S15,

$$L_{d,n} \approx \frac{2(1-\alpha)V_{b,n}}{\pi R_0^2} + \left(\frac{4}{3}R_0 + \frac{2\alpha V_0}{\pi R_0^2}\right) + \frac{2\alpha V_0}{\pi R_0^2}\varsigma_{s,n} + \left(\frac{4}{3}R_0 - \frac{4V_0}{\pi R_0^2}\right)\zeta'_{R,n}. \tag{S20}$$

Next, we calculate the slope of the best linear fit of L_d vs L_b plot using Eq. S8. The covariance can be calculated using Eqs. S19 and S20.

$$Cov(L_{b,n}, L_{d,n}) = \frac{2(1-\alpha)\sigma_{vb}^2}{(\pi R_0^2)^2} + 8c\left(\frac{R_0}{3} - \frac{V_0}{\pi R_0^2}\right)^2\sigma_R^2. \tag{S21}$$

Variance in length at birth which is the denominator in Eq. S8 is,

$$\sigma_b^2 = \frac{\sigma_{vb}^2}{(\pi R_0^2)^2} + 4\left(\frac{R_0}{3} - \frac{V_0}{\pi R_0^2}\right)^2\sigma_R^2. \tag{S22}$$

The variance in volume at birth (σ_{vb}) is calculated using Eqs. S1 and S16,

$$\sigma_{vb}^2 = \frac{\alpha^2\sigma_{bd}^2 + 4\sigma_m^2}{\alpha(2-\alpha)}V_0^2. \tag{S23}$$

Hence, we find the slope to be,

$$m_{bd} = \frac{2(1-\alpha)\frac{\alpha^2\sigma_{bd}^2+4\sigma_m^2}{\alpha(2-\alpha)}\frac{V_0^2}{(\pi R_0^2)^2} + 8c\left(\frac{R_0}{3} - \frac{V_0}{\pi R_0^2}\right)^2 \sigma_R^2}{\frac{\alpha^2\sigma_{bd}^2+4\sigma_m^2}{\alpha(2-\alpha)}\frac{V_0^2}{(\pi R_0^2)^2} + 4\left(\frac{R_0}{3} - \frac{V_0}{\pi R_0^2}\right)^2 \sigma_R^2}. \quad \text{(S24)}$$

At $n + k^{th}$ generation, the length at birth follows from Eq. S19,

$$L_{b,n+k} \approx \frac{V_{b,n+k}}{\pi R_0^2} + \frac{2}{3}R_0 + \left(\frac{2}{3}R_0 - \frac{2V_0}{\pi R_0^2}\right)\zeta'_{R,n+k-1}. \quad \text{(S25)}$$

We want to find the Pearson correlation coefficient between length at birth in n^{th} and $(n+k)^{th}$ generation. The covariance between the two variables is,

$$Cov(L_{b,n}, L_{b,n+k}) = \frac{Cov(V_{b,n}, V_{b,n+k})}{(\pi R_0^2)^2} + 4\left(\frac{R_0}{3} - \frac{V_0}{\pi R_0^2}\right)^2 Cov(\zeta'_{R,n-1}, \zeta'_{R,n+k-1}). \quad \text{(S26)}$$

The two covariance terms on the right side of Eq. S26 can be calculated using Eq. S1 and Eq. S18. Substituting the covariance expressions into Eq. S26 and calculating the Pearson correlation coefficient using Eq. S12,

$$r = \frac{(1-\alpha)^k\frac{\alpha^2\sigma_{bd}^2+4\sigma_m^2}{\alpha(2-\alpha)}\frac{V_0^2}{(\pi R_0^2)^2} + 4c^k\left(\frac{R_0}{3} - \frac{V_0}{\pi R_0^2}\right)^2 \sigma_R^2}{\frac{\alpha^2\sigma_{bd}^2+4\sigma_m^2}{\alpha(2-\alpha)}\frac{V_0^2}{(\pi R_0^2)^2} + 4\left(\frac{R_0}{3} - \frac{V_0}{\pi R_0^2}\right)^2 \sigma_R^2}. \quad \text{(S27)}$$

The value of r decreases for increasing k as verified using the adder model simulations (Figure S4A). For $\sigma_R << \frac{\alpha^2\sigma_{bd}^2+4\sigma_m^2}{\alpha(2-\alpha)}$, we find that $r(k) \approx (1-\alpha)^k$. Hence, in this regime, we regain the correlation formula for the case where radius variability vanishes. This is evident in Figure S4B where the correlation approaches $(1-\alpha) = 0.5$ for the adder model ($\alpha = 0.5$) when σ_R is small compared to $\frac{\alpha^2\sigma_{bd}^2+4\sigma_m^2}{\alpha(2-\alpha)}$.

S1.2.1 Cell width for a particular lineage is fixed.

Similar to the model in Section S1.1, we can test a volume sizer (α=1) model where the cell width is fixed in a particular lineage ($c = 1$). Substituting $\alpha = c = 1$ in Eq. S27, we get,

$$r = \frac{4\left(\frac{R_0}{3} - \frac{V_0}{\pi R_0^2}\right)^2 \sigma_R^2}{\frac{\alpha^2\sigma_{bd}^2 + 4\sigma_m^2}{\alpha(2-\alpha)}\frac{V_0^2}{(\pi R_0^2)^2} + 4\left(\frac{R_0}{3} - \frac{V_0}{\pi R_0^2}\right)^2 \sigma_R^2}. \tag{S28}$$

Although the exact expression differs from the previous section, we find that the correlation between birth lengths k generations apart is independent of k, similar to the previous section and contrary to experimental results.

S1.2.2 Using conditional correlations to estimate α

We aim to find the underlying volume regulation strategy despite the cell width fluctuations. In the main text, we explain using a graphical representation (Figure 3B), the reason for using the conditional correlation $(L_{b,n}, L_{d,n})|(R_{n-1}, R_n)$ for that purpose. In this section, we explain the method based on the model motivated in the previous section. Then, we estimate the accuracy of the method to elucidate the underlying division strategy.

Eqs. S19 and S20 provide an expression for the birth and division lengths, respectively. Rearranging the radius fluctuation terms, we obtain,

$$L_{b,n} - \frac{2V_0}{\pi R_0^2} - \left(\frac{2}{3} - \frac{2V_0}{\pi R_0^3}\right) R_{n-1} \approx \frac{V_{b,n}}{\pi R_0^2}, \tag{S29}$$

$$L_{d,n} - \left((2+\alpha)\frac{2V_0}{\pi R_0^2}\right) - \left(\frac{4}{3}R_0 - \frac{4V_0}{\pi R_0^2}\right) R_n \approx \frac{2(1-\alpha)V_{b,n}}{\pi R_0^2} + \frac{2\alpha V_0}{\pi R_0^2}\zeta_{s,n}. \tag{S30}$$

The term on the left side of Eq. S29 corresponds to the residual $(L_{b,n}|(R_{n-1}, R_n))$ obtained on linear regression of $L_{b,n}$ on R_{n-1} and R_n. Similarly, the left side of Eq. S30 corresponds to the residual $(L_{d,n}|(R_{n-1}, R_n))$ obtained by regressing $L_{d,n}$ instead of $L_{b,n}$. The slope of

the best linear fit of $L_{d,n}|(R_{n-1}, R_n)$ vs $L_{b,n}|(R_{n-1}, R_n)$ plot is $2(1-\alpha)$. Thus, this method can be used to obtain the division strategy parameter, α.

The expressions in Eqs. S29 and S30 assume that there is no contribution to the cell width fluctuations from measurement noise. However, the measured cell radius ($R_{t,n}$) in the experiments is a combination of the actual radius (R_n) and measurement noise in radius ($\zeta_{Re,n}$). Assuming an additive measurement noise in cell radius, we obtain the measured cell radius to be,

$$R_{t,n} = R_0 + c(R_{n-1} - R_0) + \sqrt{1-c^2}R_0\zeta_{R,n} + \zeta_{Re,n}. \tag{S31}$$

We assume that $\zeta_{Re,n}$ is normally distributed with zero mean and standard deviation $=$ $\sigma_{Re}R_0$. It is also assumed to be independent of R_n but it can be correlated with $\zeta_{Re,n-1}$, i.e., $\langle\zeta_{Re,n-1}\zeta_{Re,n}\rangle = c_e$. Using Eq. S31, we obtain the CV in measured cell radius to be (σ_{Rt}),

$$\sigma_{Rt}^2 = \sigma_R^2 + \sigma_{Re}^2. \tag{S32}$$

The correlation between radius in consecutive generations is,

$$c_t = \frac{c\sigma_R^2 + c_e\sigma_{Re}^2}{\sigma_R^2 + \sigma_{Re}^2}. \tag{S33}$$

We wanted to test whether the value of α calculated using the conditional correlation $((L_{b,n}, L_{d,n})|(R_{n-1}, R_n))$ is accurate for different values of σ_{Re} and c_e. We vary the value of σ_{Re} and c_e between 0 to σ_{Rt} and 0 to 1, respectively, in the simulations of the model shown in Figure 3A of the main text. Further, we restricted ourselves to those values of σ_{Re} and c_e for which correlation c was between 0 and 1. We measure the cell volumes, and lengths at birth and division, and the cell radius. We plot the difference between the α values calculated using the correlation between birth and division volumes, and the conditional correlation $((L_{b,n}, L_{d,n})|(R_{n-1}, R_n))$. We find that the difference between the two alpha values is small

- within 0.2 of each other for various parameters obtained from *E. coli* experiments in Ref. [S3] (Figures S5A, S5E).

Next, we wanted to test that the conditional correlation $((L_{b,n}, L_{d,n})|(R_{n-1}, R_n))$ is a suitable method to probe cell cycle regulation mechanisms regardless of the underlying cell cycle model. Hence, we tested the conditional correlation method on cell regulation strategies other than the model where the cell division volume is solely determined by the birth volume. In these cell cycle models, the size at the initiation of DNA replication also determines the division size [S1, S2, S5, S13]. We tested two previously proposed models (the CH model and the parallel adder model) where DNA replication solely controls cell division. In one model (CH model), cell division happens after a certain average time from the initiation of DNA replication [S2, S14]. In the parallel adder model, division happens after a certain volume per origin is added from the initiation volume [S13, S15]. Another strategy called the concurrent processes model was also tested where both DNA replication and cell birth control the cell division volume. In this model, cell division happens when the slowest of the two processes - 1. a certain time from initiation of DNA replication elapses, 2. after a particular volume from birth is added- is completed [S1, S5]. In the simulation of these models, we track the birth and division volumes and lengths, and the cell width for multiple generations and lineages. To test the accuracy of the conditional correlation $(L_{b,n}, L_{d,n})|(R_{n-1}, R_n)$ as an indicator of the division volume control strategy in these models, we find the difference between two correlation values - 1. $V_{b,n}$ and $V_{d,n}$, 2. conditional correlation between $L_{b,n}$ and $L_{d,n}$ upon fixing R_{n-1} and R_n. The difference between the two correlations for different values of σ_{Re} and c_e is small - again within 0.1 of each other (Figures S5B-S5D).

Thus, the conditional correlation $(L_{b,n}, L_{d,n})|(R_{n-1}, R_n)$ is an appropriate method to find the underlying cell cycle strategy controlling division volume using cell length data and despite cell width fluctuations.

S2 Data analysis

S2.1 Analyzing experimental data

We analyzed mother-machine data from Refs. [S3], [S6], and [S5]. These datasets were chosen as they had single-cell width measurements along with cell lengths at birth and division. We restricted our analysis to those cells for which we could find the daughter and granddaughter cells. Additionally, only for the experimental datasets in Ref. [S3], we removed the cells whose radii were outliers. These cells appeared as smaller separate islands in the R_n vs R_{n-1} plot and could be identified visually. We used these cells for the calculation of model parameters for six different growth conditions of Ref. [S3] (Table S3) and to find the correlation and conditional correlation involving birth lengths, division lengths, and cell radius (Table S1, Table S2, Table 1 and Figure 4 of the main text).

We used Fisher's z transformation to calculate the 95% confidence intervals of the Pearson correlation coefficient, r [S16]. Using the z value $= \frac{1}{2}\ln(\frac{1+r}{1-r})$, the 95% confidence intervals for z are $\left[z - \frac{1.96}{\sqrt{N-3}}, z + \frac{1.96}{\sqrt{N-3}}\right]$ for number of measurements (N) being large. The confidence intervals in correlation space are calculated by back-transforming the confidence intervals of z (CI_z). Mathematically, the 95% confidence intervals for the Pearson correlation coefficients in the paper are equal to $\frac{e^{2CI_z}-1}{e^{2CI_z}+1}$.

S2.2 Estimating model parameters

In the section "Implications on cell width fluctuations" of the main text, we substitute model parameters into Eq. 3 of the main text to calculate the correlation between birth lengths in successive generations (Table S3). In this section, we explain the methodology to determine various model parameters.

The model parameters were estimated using experimental data from Ref. [S3]. The mean radius (R_0), and CV (σ_R) are the average and CV of the measured radius in a particular

experimental dataset. c is the Pearson correlation coefficient of the cell radius between mother and daughter cells. The average volume at birth (V_0) is $\pi R_0^2 \langle L_b \rangle - \frac{2}{3}\pi R_0^3$, where $\langle L_b \rangle$ is the mean length at birth. σ_m is the standard deviation of the division ratio which is the ratio of the length at birth in the daughter cell and length at division in the mother cell. α is calculated using the slope of the best linear fit of $L_{d,n}|(R_{n-1}, R_n)$ vs $L_{b,n}|(R_{n-1}, R_n)$ plot. Using the estimates of these parameters and Eq. S22, we can calculate the standard deviation of division size noise, σ_{bd}. Note that in one of the seven conditions studied in Ref. [S3], we obtain a complex value for σ_{bd}. We do not show that dataset in Table S3.

S3 Simulations

Simulations were carried out using MATLAB R2021b.

In Figure 2 of the main text, and Figure S1, we carried out simulations of the "Pole formation after division" model. The parameters are the same as in Ref. [S4]. The cell division strategy being used was a volume sizer with the critical volume for division being $2V_0 = 3.77\mu m^3$. 10000 cell lineages were initiated with a particular division volume drawn from a normal distribution with mean size $2V_0$ and CV = 0.065. Each cell lineage was simulated for 25 generations. The cell radius R for each lineage was drawn from a normal distribution with mean = $0.55\mu m$ and CV = 0.035 and it stayed constant over the 25 generations. The results do not change on simulating over 50 generations. The length at division is calculated based on a spherocylindrical cell geometry in Figure 2A of the main text. Upon division, the length at birth, division, and radius were noted for the cell. A measurement error, drawn from a normal distribution with mean 0 and standard deviation = 0.04 μm, was added to each length measurement. Upon division, we tracked one of the two daughter cells in the next generation. The cells divided, on average, symmetrically by length with a CV = 0.032. Due to the hemispherical pole formation at mid-cell after birth,

$\frac{R}{3}$ was added to the length at birth to keep the total volume conserved.

In Figure 2 of the main text, we simulate the "Pole formation before division" model. We have the same initial conditions and parameters as the "Pole formation after division" model. The difference is in the calculation of birth and division lengths. We assume that there are two fully formed cells just before division (Figure S2A). Also, the cell divides, on average, symmetrically by volume with CV = 0.032. We also test the same model with the cell dividing symmetrically by length on average in Figure S3.

In Figure 3 of the main text, and Figure S4, we simulate the model "Changing radii in a lineage" (Figure 3A). The model parameters are determined using the experimental data in the fastest growth condition ($T_d = 17$ min) of Ref. [S3]. 10000 cells are initialized with a division volume drawn from a normal distribution with mean, $2V_0 = 5.54\mu m^3$ and CV = 0.19. The cells have a radius drawn from a normal distribution with mean = 0.49 μm and standard deviation = 0.0765. Each lineage is simulated over 25 generations. The radii are correlated (Pearson correlation coefficient = 0.86) between consecutive generations and have the same mean (= 0.49 μm) and CV (= 0.0765) as the initial cells. In Figure S4B, we vary the CV of cell radius to be between 0 and 0.13. The division and birth lengths are determined as stated in Figure 3A of the main text. The cell divides, on average, symmetrically by volume (CV = 0.06). The division volume is set by an adder model from cell birth with mean volume added = 2.77 μm^3. The size additive division volume noise has a normal distribution with mean = 0 and standard deviation = 0.53 μm^3. The birth and division lengths have an additive measurement error which is drawn from a normal distribution with mean = 0 and standard deviation = 0.04 μm.

In Figure S5A-S5D, we wanted to verify that the conditional correlation method put forth in Section S1.2.2 is an appropriate method to study the cell cycle regulation mechanisms even when the cell radius is not accurately determined. Thus, we fix the CV of the measured cell radii = (0.0765) and the correlation between the radii in consecutive generations (= 0.86).

We vary the contribution of the measurement error to the radius CV and the correlation between the measurement errors in radii measurements of consecutive generations. The actual radius CV and the correlation between the actual radii in consecutive generations are obtained using Eqs. S32 and S33, respectively. Each plot in Figure S5 is obtained using simulations of a different cell cycle model. In Figure S5A, we follow the simulation procedure as mentioned in the previous paragraph ("Changing radii in a lineage" model). In Figure S5B, we plot the simulation results of the concurrent processes model. The division event is controlled by the slowest of the two processes - 1. The addition of a particular volume ($= 1.10\ \mu m^3$) from birth. A size additive division noise, drawn from a normal distribution, is added to the adder model from birth (mean = 0; standard deviation = $0.2\ \mu m^3$), 2. The division happens after a time = 55 min from the initiation of DNA replication. We add a time additive division noise drawn from a normal distribution with mean = 0 and standard deviation = 12 min. In Figure S5C, we plot the results from the CH model simulations. In this model, division happens after a time T from the initiation of DNA replication. T is drawn from a normal distribution with mean = 60 min and CV = 0.05. For Figure S5D, we simulate the parallel adder model where the division event occurs after the addition of Δ_{id} volume per origin from the initiation of DNA replication. Δ_{id} is drawn from a normal distribution with mean = $0.5\ \mu m^3$ and CV = 0.1. For all of the models, the initiation of DNA replication happened upon the addition of Δ_{ii} volume per origin from the initiation in the previous cell cycle. Δ_{ii} is drawn from a normal distribution with mean = $0.69\ \mu m^3$, CV = 0.1 for the concurrent processes model; mean = 0.7, CV = 0.15 for the CH model and mean = 0.65, CV = 0.2 for the parallel adder model. In the simulations, 10000 cells were initiated at birth with a volume = $0.6545\ \mu m^3$. Each cell grew exponentially with a different growth rate drawn from a normal distribution with mean = $0.0107\ min^{-1}$ and CV = 0.1. Upon division, lengths at birth and division, and the cell radius were noted with measurement errors. For the length measurements, the error was additive and it was drawn

from a normal distribution with mean = 0 and standard deviation = 0.04 μm. We tracked one of the two daughter cells over 25 generations for each lineage. The cells divided, on average, symmetrically in volume with a standard deviation in division ratio = 0.03. The same parameters are also used for the simulations in Figure S7. For Figure S5E, we follow the same simulation model as in Figure S5A but we use different parameters. The parameters are shown in Table S3.

S4 Cell controls surface area instead of volume

Till now we have discussed scenarios where the cell cycle control is on cell volume. However, if we assume that biomass is the relevant quantity being controlled, we need to find a cell geometry characteristic that is most related to biomass accumulation. Ref. [S17] observed that biomass growth is proportional to cell surface area growth during a cell cycle, hence, cell surface area could be a suitable proxy for it. In this section, we discuss a case where the cell surface area is being regulated. We find that cell width fluctuations have a similar effect on the correlations between the lengths at birth and division, and birth lengths k generations apart.

We assume that the cell divides when it reaches a surface area S_d which is solely determined by its surface area at birth,

$$S_d = 2(1-\alpha)S_b + 2\alpha S_0(1+\zeta_s(0,\sigma_{bd})). \tag{S34}$$

The equation is similar to Eq. S1 with cell volume replaced with cell surface area.

Assuming that the cell is a spherocylinder, the total surface area of the cell is always related to its length as,

$$S = 2\pi RL. \tag{S35}$$

The above equation holds even for a cell undergoing constriction at the mid-cell. The length at division in generation n and the birth length in generation $n+1$ is as follows,

$$L_{d,n} = \frac{S_{d,n}}{2\pi R_n}, \tag{S36}$$

$$L_{b,n+1} = \frac{S_{b,n+1}}{2\pi R_n}. \tag{S37}$$

We follow the same assumptions as Section S1.2 to model the cell width fluctuations. We assume that cells do not change their radius when they divide. However, the radius changes during the cell cycle such that the radius when the cell divides in generation $n+1$ is related to the radius in generation n as stated in Eq. S18. The cell divides symmetrically by surface area on average with the standard deviation in division ratio being σ_m as before. Since cell length is proportional to the surface area, the length division ratio has the same mean and standard deviation.

Assuming the noise in radius ($\zeta_{R,n}$), division ratio (δ_n), and division size noise ($\zeta_{s,n}$) to be small, we obtain, till first order, the expressions for the birth and division lengths in generation n,

$$L_{b,n} \approx \frac{S_{b,n}}{2\pi R_0} - \frac{S_0}{2\pi R_0}\zeta'_{R,n-1}, \tag{S38}$$

$$L_{d,n} \approx \frac{2(1-\alpha)S_{b,n}}{2\pi R_0} + \frac{2\alpha S_0}{2\pi R_0} + \frac{2\alpha S_0}{2\pi R_0}\zeta_{s,n} - \frac{2S_0}{2\pi R_0}\zeta'_{R,n}, \tag{S39}$$

where $\zeta'_{R,n} = c\frac{R_{n-1}-R_0}{R_0} + \sqrt{1-c^2}\zeta_{R,n}$, as previously defined. Using Eqs. S38 and S39, we obtain the slope of the best linear fit of L_d vs L_b plot to be,

$$m_{bd} = \frac{2(1-\alpha)\frac{\alpha^2\sigma_{bd}^2+4\sigma_m^2}{\alpha(2-\alpha)} + 2c\sigma_R^2}{\frac{\alpha^2\sigma_{bd}^2+4\sigma_m^2}{\alpha(2-\alpha)} + \sigma_R^2}. \tag{S40}$$

The correlation between birth lengths k generations apart is,

$$r(k) = \frac{(1-\alpha)^k \frac{\alpha^2\sigma_{bd}^2 + 4\sigma_m^2}{\alpha(2-\alpha)} + c^k \sigma_R^2}{\frac{\alpha^2\sigma_{bd}^2 + 4\sigma_m^2}{\alpha(2-\alpha)} + \sigma_R^2}. \tag{S41}$$

Eq. S41 is similar to Eq. 3 of the main text assuming that $\frac{\pi R_0^3}{3} << V_0$. There are two contributions to the length correlations - one from cell surface area regulation strategy, $f(S_b)$, and the other from correlated radii across generations. The same arguments that were presented in the case of cell volume regulation in the main text will apply here for calculating α and estimating the intrinsic cell width variability. Hence, our analysis points to width fluctuations having the same effect on length correlations regardless of the cell characteristics (surface area or volume) under regulation.

S5 Analysis of fission yeast data

We applied the conditional correlation tests to the WT single-cell fission yeast (*Schizosaccharomyces pombe*) data in Facchetti *et al.* [S4]. Fission yeast is hypothesized to follow a birth-to-division sizer i.e., the cell divides when it reaches a particular surface area independent of the surface area at birth. Since cell length is proportional to surface area for rod-shaped organisms such as fission yeast and *E. coli*, cell length is often used as a proxy for the surface area. However, Facchetti *et al.* and our work show that inference of cell cycle regulation strategy using cell lengths could be confounded by fluctuations in cell width when the actual size control is on other cell characteristics such as cell surface area/volume.

Hence, the correlation between the length at birth (L_b) and the length at division (L_d) is expected to be non-zero for fission yeast (Figure S8A). Controlling for the radius fluctuations would render the conditional correlation zero. However, we find that the conditional correlation between the lengths at birth and division upon conditioning on the cell radii remains

almost the same in experimental data (Figure S8B).

Facchetti *et al.* used a different method to estimate the conditional correlation. In this method, they restricted the radius values to be within a small range around the mean. The range value is monotonically related to the radius variability. Conditional correlation $r(L_b, L_d|R)$ is the correlation between L_b and L_d in the limiting case where the range is zero. In fission yeast, it was predicted that the slope of the plot L_d versus L_b would decrease as the range / radius variability decreased. However, for WT data, the slope decreased slightly (from 0.24 for range = 0.3 μm to 0.2 for range = 0.06 μm). Our results in Figure S8 are consistent with these values. These results point to fission yeast being a near-sizer. Using fission yeast mutants that had greater radius variability than WT, it was found in Ref. [S4] that the slope increased in accordance with the prediction. However, to show that the slope is indeed zero (sizer) for negligible radius variability, more precise measurements are needed.

We also analyzed the *E. coli* data presented in the main text of the paper [S3] using the method in Facchetti *et al.*. We found that the slope values remained constant or increased with decreasing range for all six growth conditions (Figure S9). Furthermore, the slope values were close to 1 which gives additional evidence that the cell size regulation strategy in *E. coli* agrees with an adder rather than a sizer.

Supporting References

S1. Micali, G., Grilli, J., Osella, M., and Lagomarsino, M.C. (2018). Concurrent processes set *E. coli* cell division. Science Advances *4*, eaau3324.

S2. Ho, P.Y. and Amir, A. (2015). Simultaneous regulation of cell size and chromosome replication in bacteria. Frontiers in Microbiology *6*, 662.

S3. Taheri-Araghi, S., Bradde, S., Sauls, J.T., Hill, N.S., Levin, P.A., Paulsson, J., Vergas-

sola, M., and Jun, S. (2015). Cell-size control and homeostasis in bacteria. Current biology *25*, 385–391.

S4. Facchetti, G., Knapp, B., Chang, F., and Howard, M. (2019). Reassessment of the basis of cell size control based on analysis of cell-to-cell variability. Biophysical Journal *117*, 1728–1738.

S5. Colin, A., Micali, G., Faure, L., Lagomarsino, M.C., and van Teeffelen, S. (2021). Two different cell-cycle processes determine the timing of cell division in *Escherichia coli.* eLife *10*, e67495.

S6. Si, F., Le Treut, G., Sauls, J.T., Vadia, S., Levin, P.A., and Jun, S. (2019). Mechanistic origin of cell-size control and homeostasis in bacteria. Current Biology *29*, 1760–1770.

S7. Kar, P., Tiruvadi-Krishnan, S., Männik, J., Männik, J., and Amir, A. (2021). Distinguishing different modes of growth using single-cell data. eLife *10*, e72565.

S8. Eun, Y.J., Ho, P.Y., Kim, M., LaRussa, S., Robert, L., Renner, L.D., Schmid, A., Garner, E., and Amir, A. (2018). Archaeal cells share common size control with bacteria despite noisier growth and division. Nature Microbiology *3*, 148–154.

S9. Amir, A. (2014). Cell size regulation in bacteria. Physical Review Letters *112*, 208102.

S10. Margolin, W. (2009). Sculpting the bacterial cell. Current Biology *19*, R812–R822.

S11. Raskin, D.M. and De Boer, P.A. (1999). Rapid pole-to-pole oscillation of a protein required for directing division to the middle of *Escherichia coli.* Proceedings of the National Academy of Sciences *96*, 4971–4976.

S12. Hu, Z. and Lutkenhaus, J. (1999). Topological regulation of cell division in *Escherichia coli* involves rapid pole to pole oscillation of the division inhibitor MinC under the control of MinD and MinE. Molecular Microbiology *34*, 82–90.

S13. Witz, G., van Nimwegen, E., and Julou, T. (2019). Initiation of chromosome replication controls both division and replication cycles in *E. coli* through a double-adder mechanism. eLife *8*, e48063.

S14. Cooper, S. and Helmstetter, C.E. (1968). Chromosome replication and the division cycle of *Escherichia coli* Br. Journal of Molecular Biology *31*, 519–540.

S15. Logsdon, M.M., Ho, P.Y., Papavinasasundaram, K., Richardson, K., Cokol, M., Sassetti, C.M., Amir, A., and Aldridge, B.B. (2017). A parallel adder coordinates mycobacterial cell-cycle progression and cell-size homeostasis in the context of asymmetric growth and organization. Current Biology *27*, 3367–3374.

S16. Nicholls, A. (2014). Confidence limits, error bars and method comparison in molecular modeling. Part 1: the calculation of confidence intervals. Journal of Computer-Aided Molecular Design *28*, 887–918.

S17. Oldewurtel, E.R., Kitahara, Y., and van Teeffelen, S. (2021). Robust surface-to-mass coupling and turgor-dependent cell width determine bacterial dry-mass density. Proceedings of the National Academy of Sciences *118*, e2021416118.
